# Propensity score‐adjusted three‐component mixture model for drug‐drug interaction data mining in FDA Adverse Event Reporting System

**DOI:** 10.1002/sim.8457

**Published:** 2019-12-27

**Authors:** Xueying Wang, Lang Li, Lei Wang, Weixing Feng, Pengyue Zhang

**Affiliations:** ^1^ Institute of Intelligent System and Bioinformatics, College of Automation, Harbin Engineering University Harbin China; ^2^ Department of Biomedical Informatics College of Medicine, The Ohio State University Columbus Ohio

**Keywords:** adverse drug event, drug‐drug interaction, false discovery rate, FDA adverse event reporting system, propensity score

## Abstract

With increasing trend of polypharmacy, drug‐drug interaction (DDI)‐induced adverse drug events (ADEs) are considered as a major challenge for clinical practice. As premarketing clinical trials usually have stringent inclusion/exclusion criteria, limited comedication data capture and often times small sample size have limited values in study DDIs. On the other hand, ADE reports collected by spontaneous reporting system (SRS) become an important source for DDI studies. There are two major challenges in detecting DDI signals from SRS: confounding bias and false positive rate. In this article, we propose a novel approach, propensity score‐adjusted three‐component mixture model (PS‐3CMM). This model can simultaneously adjust for confounding bias and estimate false discovery rate for all drug‐drug‐ADE combinations in FDA Adverse Event Reporting System (FAERS), which is a preeminent SRS database. In simulation studies, PS‐3CMM performs better in detecting true DDIs comparing to the existing approach. It is more sensitive in selecting the DDI signals that have nonpositive individual drug relative ADE risk (NPIRR). The application of PS‐3CMM is illustrated in analyzing the FAERS database. Compared to the existing approaches, PS‐3CMM prioritizes DDI signals differently. PS‐3CMM gives high priorities to DDI signals that have NPIRR. Both simulation studies and FAERS data analysis conclude that our new PS‐3CMM is a new method that is complement to the existing DDI signal detection methods.

## 1 INTRODUCTION

Drug‐drug interaction (DDI) can change either the pharmacokinetics or pharmacodynamics (PD) drug response when two or more drugs are coadministered together.^1^ Clinically, adverse drug events (ADEs) are the primary safety concern of DDIs. The incidence of DDI‐induced ADEs is expected to increase due to the increasing trend of polypharmacy. Currently in US, 21.8% of US population receives three or more prescription drugs simultaneously, and 10.7% of the persons even have five or more.^2^ Comparatively, these percentages were only 11.0% and 3.6% before 2000.^2^ DDI‐induced ADEs have become a major challenge to the clinical care setting, as DDIs are suspected to be related with up to 59.1% of ADEs, and they became a common cause for drugs withdraw from the market.^3^, ^4^ However, only limited DDIs have been detected in premarketing clinical trials; most of these trials focus on single‐drug effect, and comedications due to the comorbidities are usually limited by stringent inclusion/exclusion criteria. Thus, most DDIs are discovered through analyzing the postmarketing pharmacovigilance databases.^5^

The spontaneous reporting system (SRS) is an important type of pharmacovigilance database for the postmarketing ADE detection.^5^ SRS collects ADE reports from a variety of sources including patients, pharmaceutical companies, and healthcare professionals. As the SRS collects only “cases”, ADE incidence rates are usually inflated. Despite the potential bias, it is commonly acceptable to use the SRS and test whether a drug or a drug combination has an increased ADE relative to the other patients who did not have this drug or drug combination. This analysis is also called disproportionality analyzes (DPAs) for the drug‐induced ADE signal detection. DPAs are originally developed to detect single‐drug‐ADE signals by comparing the observed report frequency for a drug‐ADE combination (ie, observed frequency) to the baseline frequency under the no association assumption (ie, expected frequency).^6^ Well‐known DPAs include the Information Component (IC),^7^ IC with Dirichlet prior,^8^ empirical Bayes geometric mean (EBGM) also known as the multigamma Poisson shrinkage (MGPS),^9^ and the three‐component mixture model (3CMM).^10^ On the other hand, the likelihood ratio test (LRT) and its extension zero‐inflated Poisson model‐based LRT (ZIP‐LRT) are able to test the associations between an ADE and many drugs.^11^, ^12^

The DPA method has also been used for investigating the DDI‐induced ADEs by comparing the observed report frequency of a drug‐drug‐ADE combination to its baseline frequency assuming no interactions between two drugs. By considering the drug‐drug pair as a “new drug”, the observed report frequency can be obtained from the SRS database. Therefore, the single‐drug‐ADE signals detection methods can be extended to identify the DDI signals by designing a new baseline frequency. For example, by extending the baseline frequency from a two‐item set to the three‐item set based on a so‐called concept of “market basket problem”, the EBGM approach was extended to identify the suspected DDIs.^13^, ^14^ The IC with Dirichlet prior approach was extended for detecting DDI signals based on a proposed three‐order IC function.^8^ The three‐order IC is defined as the deviation between the IC under the condition of the third event and the original IC, and the baseline frequency account for both the main effects and the two‐drug interactions. Despite these extensions of DPA methods, Thakrar et al^15^ proposed multiplicative and additive models to detect DDI signals in the FDA Adverse Event Reporting System (FAERS) database. Under the assumption of no interaction, the multiplicative model assumes that the risk associated with one drug multiplies to the background risk, and the relative risk (RR) associated with this drug is not influenced by the other drug; the additive model assumes that the risk associated with one drug is additive to the background risk, and this drug's risk is not influenced by the other drug. Norén et al^16^ also proposed a well‐known Ω shrinkage method for the suspected DDIs detection in individual case safety reports (ICSRs) data. It determines the baseline model with an addictive model and calculates a shrinkage observed‐to‐expected ratio of the ADE risk for the drug‐drug pair. The shrinkage penalizes the signals of rare drug‐drug‐ADE combinations with a prespecified parameter.

Detecting DDI signals from SRS has several major challenges. SRS analysis subjects to confounding bias from thousands of comedications. In single‐drug‐ADE association analysis, Dumouchel et al^9^ proposed a stratified expectation by using the Mantel‐Haenszel formula.^17^ This approach calculates the expected frequency by using the expected frequency within each strata. The stratified approach can be generalized for LRT, IC, and their aforementioned extensions as well. However, stratification is limited by the number of suspected confounders. As the increased number of strata yields reduced sample size within each strata, the power to detect DDI signals is reduced.^18^ Besides stratification, confounding bias can be addressed by multiple regression models in which confounding variables (eg, demographic variables and comedications) are treated as covariates. For example, van Puijenbroek et al^19^ proposed a multiple logistic regression model for detecting DDI signals. Though multiple regression approach is not necessarily limited by the sample size challenge, techniques for properly selecting confounding variables are still underdeveloped.^20^ False positive control is another significant challenge in detecting DDI signals from hundreds of thousands drug pairs.^10^ False discovery rate (FDR) is more practical than traditional hypothesis testing techniques (eg, *P*‐value) for large‐scale DDI signals detection. For instance, Bayesian FDR and local FDR were proposed to control false positive rate for detecting single‐drug signals.^21^, ^22^ While these approaches have not been developed for DDI signals detection.

In this article, our aim is to detect suspected DDI signals with a low FDR from the FAERS, which is a prominent SRS database. Specifically, we propose a novel approach to compute the expected report frequency, in which confounding bias is adjusted by using the comedication information. Then, we model the observed and expected frequencies for the drug‐drug‐ADE combinations under an empirical Bayes model frame work. By defining the null risk (eg, RR = 1), the proposed approach estimated local FDR for each signal. The rest of the article is organized as follow. In Section 2, we review the Ω shrinkage method and describe the proposed method. In Section 3, we conduct simulation studies to evaluate and compare the performance of the proposed approach and the Ω shrinkage method. In Section 4, we illustrate the utilization of the proposed approach by analyzing the FAERS. Last, Section 5 concludes and discusses the proposed approach.

## 2 MATERIALS AND METHODS

### 2.1 The FAERS database and data processing

The FAERS is a prominent SRS maintained by the U.S. Food and Drug Administration. The FAERS database collects adverse event reports directly from manufacturers, pharmacists, physicians, nurses, and consumers.^23^ Each report consists of information including: (1) patients demographics; (2) drugs information which classified as “primary suspect”, “secondary suspect”, “concomitant”, and “interacting”; (3) ADEs coded by using the Medical Dictionary for Regulatory Activities' (MedDRA) Preferred Terms (PTs)^24^; (4) patient outcomes; (5) report sources; and (6) indications coded by using MedDRA PT code for the reported drugs. For FAERS analysis, the ADEs can be defined by using MedDRA PTs. However, the drug names may involve abbreviations, synonyms, brand names, and spelling mistakes. Thus, normalization of the drug names is necessary for FAERS analysis. The FAERS database has two major advantages: (1) massive sample size (>18 million reports)^25^ and (2) the implementation of MedDRA PT allows the ADEs to be directly available. Despite the advantages, the FAERS database also has some limitations. First, the true ADE incidence rates cannot be obtained, as the FAERS database only contains ADE reports. Second, there is a great uncertainty between the drugs and the ADEs on a report, as the report collects all possible drugs and ADEs for a patient.^9^ Third, the quality of the FAERS database may be compromised by inaccurate reports and missing data (eg, age and gender). For instance, over 30% of the FAERS reports involve missing data, as reporting of demographics is voluntary.^26^ Although the FAERS database has the aforementioned limitations, it is a valuable source for DDI study.

In this article, we selected FAERS reports from 2004Q1 to 2012Q3 as our data source. To avoid the spurious associations caused by the duplicated reports, we only left the reports with the latest primary ID and removed other reports with the same primary IDs. As there was a great uncertainty of the drugs' roles (eg, primary suspect), we used all the drugs on each report including “primary suspect,” “secondary suspect”, “concomitant”, and “interacting”. Furthermore, the drug names were normalized into generic names by using the dictionary proposed by DrugBank and Wu et al^27^, ^28^ Frequent drug names (report frequency > 999) that failed to be normalized were manually checked and corrected. After data processing, our FAERS dataset contained 4 280 322 reports with 1180 generic drug names and 15 445 MedDRA PT ADE names. Of these 4 280 322 reports used for data analysis, about 38% of the reports involved with missing age information and nearly 10% of the reports involved with missing gender information.

**Figure 1:**
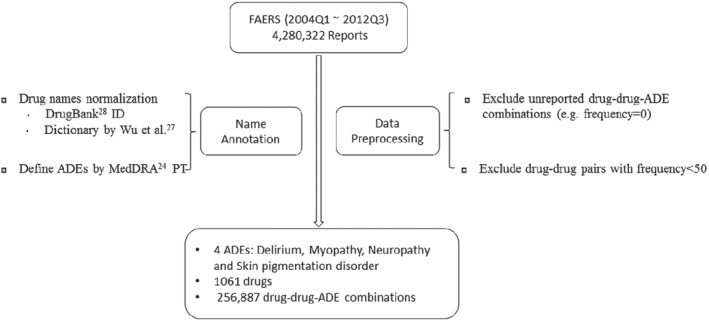
Overview of FAERS data processing

To detect potential DDI signals, we extracted the information for drug‐drug combinations and ADEs from our processed FAERS dataset. The total number of drugs in our FAERS dataset was 1180. In order to limit the search space of the drug‐drug combinations and maintain sufficient candidate drug‐drug combinations, we set criteria to filter the drug‐drug combinations by their frequencies. Specifically, all drug‐drug combinations appeared in our FAERS dataset with the frequency bigger than 50 were used in our illustrative FAERS analysis. After filtering the report frequencies of drug‐drug combinations (eg, >50), we identified 81 312 drug‐drug combinations generated by 1061 drugs. Furthermore, we selected four primary ADEs for analysis including delirium, myopathy, neuropathy, and skin pigmentation disorder.^10^ Our final dataset contained 256 887 drug‐drug‐ADE combinations which were formed from four ADEs and 1061 drugs (Figure 1).

### 2.2 Notations and definitions

For a drug‐drug‐ADE combination (eg, Drug1 and Drug2), the FAERS reports can be summarized into eight frequencies (Table 1) In Table 1, *a*, *b*, *c*, *d*, *e*, *f*, *g*, and *h* denote the eight report frequencies for a drug‐drug‐ADE combination in the FAERS, according to the status of Drug1, Drug2, and the ADE. For instance, *g* is the number of reports that contain Drug1, Drug2, and the ADE; and *h* is the number of reports that contain Drug1 and Drug2 but do not contain the ADE. Furthermore, we define the observed relative reporting rate for the ADE under the four different drug exposure status as $r_{00}=\frac{a}{a+b}$, $r_{10}=\frac{c}{c+d}$, $r_{01}=\frac{e}{e+f}$, and $r_{11}=\frac{g}{g+h}$ (Table 1). We define *N* = *g* as the observed report frequency, *E* as the expected report frequency, *λ* as the RR, and $\hat{\lambda }=\frac{N}{E}$ as the observed RR for a drug‐drug‐ADE combination. For different DPAs, *E* are calculated differently.

**Table 1:** Report frequencies and rates for a drug pair and ADE in FAERS database

Exposure status		ADE status		
Drug1	Drug2	Yes	No	Relative reporting rate
No	No	*a*	*b*	*r*_00_ = *a*/(*a* + *b*)
Yes	No	*c*	*d*	*r*_10_ = *c*/(*c* + *d*)
No	Yes	*e*	*f*	*r*_01_ = *e*/(*e* + *f*)
Yes	Yes	*g*	*h*	*r*_11_ = *g*/(*g* + *h*)

Abbreviation: ADE, adverse drug event.

### 2.3 A review of the **Ω** shrinkage method

In this section, we review the well‐known DPA to detect DDI signals from SRS by Norén et al^16^ This method, also known as the Ω shrinkage method, penalizes the signals of less frequent drug‐drug‐ADE combinations. Under this approach, based on the addictive risk model:

$$r_{11}+r_{00}=r_{10}+r_{01}$$

The expected frequency *E* is calculated as:

(1) $$E=\left[1-\frac{1}{\max\left(\frac{r_{10}}{1-r_{10}},\frac{r_{00}}{1-r_{00}}\right)+\max\left(\frac{r_{01}}{1-r_{01}},\frac{r_{00}}{1-r_{00}}\right)-\frac{r_{00}}{1-r_{00}}+1}\right]\times \left(g+h\right).$$

In Equation (1), (*g* + *h*) represents the total number of reports that contain both Drug1 and Drug2 (Table 1). The maximum functions on denominator are equivalent as *r*_10_ = max(*r*_10_, *r*_00_) and *r*_01_ = max(*r*_01_, *r*_00_). These restrictions guarantee the ADE risk caused by single drug is higher than its background risk. And the Ω shrinkage measure is calculated as

(2) $$\Omega =\log_{2}\frac{N+\alpha }{E+\alpha }.$$

In Equation (2), α is prespecified (eg, α = 0.5). The Ω shrinkage method can also be derived under the Bayesian framework by assuming *N* ∼ *Pois*(*λE*) and *λ* ∼ Γ(*α*, *α*). The posterior distribution of *λ* is also a gamma distribution: *λ* ∣ *N*, *E* ∼ Γ(*N* + *α*, *E* + *α*). To detect DDIs, the signal generation can be based on the logarithm of the lower limits of 95% credibility interval for *λ* denoted as $\;\Omega_{025}$. According to Noren et al^16^, positive $\Omega_{025}$ value indicates DDI signals.

### 2.4 Propensity score‐adjusted three‐component mixture model

Our proposed statistics for DDI signal detection is named as PS‐adjusted three‐component mixture model (PS‐3CMM). We will first introduce the calculation for the expected frequency *E* and subsequently the empirical Bayes mixture model for adjusted‐FDR estimation.

#### 2.4.1 Logistic regression‐based expected frequency for drug‐drug‐ADE combinations

If there are no confounders, logistic regression model (3) can be used to estimate the individual drug and DDI effect.

(3) $$\text{Logit}\left[P\left(\mathrm{ADE}=1\right)\right]=\beta_{0}+\beta_{1}D1+\beta_{2}D2+\beta_{3}D1D2,$$

where in Equation (3), D1 and D2 are the binary drug exposure status, P(ADE = 1) is the risk of ADE, and *β*_0_, *β*_1_, *β*_2_, *β*_3_ are the coefficients.

The estimation for the background odds of the ADE in the absence of both D1 and D2 is:

(4) $${\text{Odds}}_{00}=\frac{r_{00}}{1-r_{00}}=\exp\left({\hat{\beta }}_{0}\right).$$

Similarly, the estimations for the odds of the ADE in the absence of one of D1 and D2 are

(5) $${\text{Odds}}_{10}=\frac{r_{10}}{1-r_{10}}=\exp\left({\hat{\beta }}_{0}+{\hat{\beta }}_{1}\right),\;\text{and}$$

(6) $${\text{Odds}}_{01}=\frac{r_{01}}{1-r_{01}}=\exp\left({\hat{\beta }}_{0}+{\hat{\beta }}_{2}\right).$$

The estimation for the odds of the ADE in present of both D1 and D2 under the condition of no interaction is

(7) $${\text{Odds}}_{11}=\frac{r_{11}}{1-r_{11}}=\exp\left({\hat{\beta }}_{0}+{\hat{\beta }}_{1}+{\hat{\beta }}_{2}\right).$$

From Equations (4) to (7), we get the relation for the reporting rate (8)

(8) $$\frac{r_{11}}{1-r_{11}}\times \frac{r_{00}}{1-r_{00}}=\frac{r_{10}}{1-r_{10}}\times \frac{r_{01}}{1-r_{01}}.$$

Therefore, the baseline model can be seen as a multiplicative odds model.

From Equations (7) and (8), the expected report frequency of taking both drugs under a non‐DDI assumption can be calculated as:

(9) $$E=\left[1-\frac{1}{\frac{\frac{r_{10}}{1-r_{10}}\times \frac{r_{01}}{1-r_{01}}}{\frac{r_{00}}{1-r_{00}}}+1}\right]\times \left(g+h\right)=\frac{\exp\left({\hat{\beta }}_{0}+{\hat{\beta }}_{1}+{\hat{\beta }}_{2}\right)}{1+\exp\left({\hat{\beta }}_{0}+{\hat{\beta }}_{1}+{\hat{\beta }}_{2}\right)}\times \left(g+h\right).$$

#### 2.4.2 PS‐adjusted logistic regression‐based expected frequency for drug‐drug‐ADE combinations

Because the large number of comedication confounders cannot be straightforwardly controlled in the SRS dataset, we propose a PS‐adjusted expectation to address confounding bias. Assuming that the unobserved confounders can be characterized by the comedications in our dataset, the logistic regression (10) and the principal components (PCs) derived from comedications are used to compute the probability (ie, propensity score, PS) of taking the drug.

(10) $$\text{Logit}\left[P\left(\text{Drug}=1\right)\right]=\alpha_{0}+\sum_{i=1}^{K}\alpha_{i}\times {\mathrm{PC}}_{i}.$$

In Equation (10), *K* denote the number of PCs that we used to calculate the PS. PCs are derived from the comedications and *K* are selected by a prespecified threshold for the fraction of the total explained variance. Then, for each drug‐drug‐ADE combination, we used the logistic regression model (11) to estimate the individual drug and DDI effect with adjustment of two PSs.

(11) $$\text{Logit}\left[P\left(\mathrm{ADE}=1\right)\right]=\beta_{0}+\beta_{1}D1+\beta_{2}D2+\beta_{3}D1D2+\beta_{4}\mathrm{PS}1+\beta_{5}\mathrm{PS}2.$$

In Equation (11), D1 and D2 are the binary indicators of two drugs and PS1 and PS2 are their PSs, respectively. The expected report frequency is calculated as:

(12) $$E=\sum_{i=1}^{g+h}\frac{\mathrm{Exp}\left({\hat{\beta }}_{0}+{\hat{\beta }}_{1}+{\hat{\beta }}_{2}+{\hat{\beta }}_{4}\mathrm{PS}1_{i}+{\hat{\beta }}_{5}\mathrm{PS}2_{i}\right)}{1+\mathrm{Exp}\left({\hat{\beta }}_{0}+{\hat{\beta }}_{1}+{\hat{\beta }}_{2}+{\hat{\beta }}_{4}\mathrm{PS}1_{i}+{\hat{\beta }}_{5}\mathrm{PS}2_{i}\right)}.$$

In Equation (12), for report *i* , $\frac{\mathrm{Exp}\left({\hat{\beta }}_{0}+{\hat{\beta }}_{1}+{\hat{\beta }}_{2}+{\hat{\beta }}_{4}\mathrm{PS}1_{i}+{\hat{\beta }}_{5}\mathrm{PS}2_{i}\right)}{1+\mathrm{Exp}\left({\hat{\beta }}_{0}+{\hat{\beta }}_{1}+{\hat{\beta }}_{2}+{\hat{\beta }}_{4}\mathrm{PS}1_{i}+{\hat{\beta }}_{5}\mathrm{PS}2_{i}\right)}$ is the estimation of the PS‐adjusted probability for the ADE occurrence with only the individual drug effects. In another word, *E* is the expected report frequency of taking both drugs under a non‐DDI assumption while adjusted for potential confounders.

#### 2.4.3 FDR estimation for drug‐drug‐ADE combinations

To model the observed and expected report frequencies (ie, *N*s and *E*s), we adopt the empirical Bayes mixture model framework. As nearly 83% of report frequencies for drug‐drug‐ADE combinations in our dataset is equal to 0, we choose the three‐component empirical Bayes mixture model proposed by Zhang et al^10^ to model the RR for the drug‐drug‐ADE combinations. Moreover, the 3CMM framework also provides a much needed false positive control for the signals. We assume the drug‐drug‐ADE combinations belong to three groups. They are (i) zero DDI risk: drug‐drug‐ADE combinations have 0 frequency (eg, neither the drug combination nor the individual drugs causes the ADE); (ii) background noise: the report frequencies for drug‐drug‐ADE combinations are close to their expected frequencies due to the additive effects from two drugs; and (iii) DDI signals: drug‐drug‐ADE combinations have much higher RRs than the background additive effect.

We assume the unobserved RR (λ) follows the 3CMM (13).

$$\lambda ∼P_{1}\left\{I\left(\lambda =0\right)\right\}+\sum_{l=2}^{3}\left[P_{l}\Gamma \left(\lambda ;\alpha_{l},\beta_{l}\right)\right];$$

(13) $$\alpha_{2}=\beta_{2},\alpha_{3}>\beta_{3},\text{and}\;P_{1}+P_{2}+P_{3}=1.$$

In Equation (13), *I*(*λ* = 0) is an identity distribution that characterizes the drug‐drug‐ADE combinations with RR = 0 (eg, no ADE risk). Γ(*λ*; *α*_*l*_, *β*_*l*_) is the gamma distribution such that $\Gamma \left(\lambda ;\alpha_{l},\beta_{l}\right)=\frac{{\beta_{l}}^{\alpha_{l}}}{\Gamma \left(\alpha_{l}\right)}\lambda^{\alpha_{l}-1}\exp\left(-\beta_{l}\lambda \right)$. There are two gamma distributions with different restrictions in the 3CMM. One gamma distribution (*l* = 2) restricts *α*_2_ = *β*_2_ , and has its mean equal to 1. Therefore, this gamma distribution characterizes the drug‐drug‐ADE combinations to have a background ADE risks (ie, mean RR = 1). The other gamma distribution (*l* = 3) restricts *α*_3_ > *β*_3_ and has its mean greater than 1. Therefore, this gamma distribution characterizes drug‐drug‐ADE combinations with increased ADE risks (ie, mean RR > 1), which represents signals of DDI. Furthermore, from Equation (13), we assume *N* ∼ Pois(*λ* × *E*). Thus, the observed distribution of *N* is

(14) $$P\left(N\right)=P_{1}I\left(N=0\right)+\sum_{l=2}^{3}\left[P_{l}F\left(N;\alpha_{l},\beta_{l},E\right)\right],$$

$$\alpha_{2}=\beta_{2},\alpha_{3}>\beta_{3},\text{and}\;P_{1}+P_{2}+P_{3}=1.$$

In Equation (14), $F\left(N;\alpha_{l},\beta_{l},E\right)=\frac{\Gamma \left(N+\alpha_{l}\right)\times E^{N}\times \beta_{l}^{\alpha_{l}}}{\Gamma \left(\alpha_{l}\right)\times N!\times {\left[E+\beta_{l}\right]}^{N+\alpha_{l}}}$ is the negative binomial distribution function.

For the adverse DDIs detection, our concern is drug‐drug‐ADE combinations with positive report frequencies ( *N* > 0). Therefore, to reduce the excessive amount of drug‐drug‐ADE combination with *N* = 0, the conditional distribution (15) can be used for inference, which will not affect local FDR estimation accord to Zhang et al^10^

(15) $$P\left(N=k|\;N>0\right)=\frac{F\left(N=k;\alpha_{2},\beta_{2},E\right)+\frac{P_{3}}{P_{2}}\times F\left(N=k;\alpha_{3},\beta_{3},E\right)}{P\left(N>0;\alpha_{2},\beta_{2},N\right)+\frac{P_{3}}{P_{2}}\times P\left(N>0;\alpha_{3},\beta_{3},E\right)}\;.$$

In Equation (15), *P*(*N* > 0; *α*_2_, *β*_2_, *N*) is the probability for *N* > 0, *F*(*N* = *k*; *α*_2_, *β*_2_, *E*) is the distribution function for *P*(*N* > 0; *α*_2_, *β*_2_, *N*). The conditional log‐likelihood function for Equation (15) is:

(16) $$\mathrm{ll}\left(N;E,\left\{\alpha_{2}=\beta_{2},\alpha_{3},\beta_{3},\frac{P_{3}}{P_{2}}\right\}\right)=\sum \log\left[P\left(N=k|\;N>0\right)\right].$$

By considering $\frac{P_{3}}{P_{2}}$ as one parameter, the four parameters $\left\{\alpha_{2}=\beta_{2},\alpha_{3},\beta_{3},\frac{P_{3}}{P_{2}}\right\}$ in Equations (16) can be obtained by maximizing the observed likelihood (eg, maximum likelihood estimate). In this study, the MLEs are obtained by using the R function nlminb, which is able to conduct both unconstrained and box‐constrained optimization by using Newton‐like methods.^29^

As the mean of the first gamma component in Equation (16) equals 1, it will be served as the null distribution to compute the local FDR accord to Efron et al^22^ The local FDR for a drug‐drug‐ADE combination is defined as:

(17) $$\text{Local}\;\mathrm{FDR}\left(N=k|k>0\right)=\frac{F\left(N=k;\alpha_{2},\beta_{2},E\right)}{F\left(N=k;\alpha_{2},\beta_{2},E\right)+\frac{P_{3}}{P_{2}}\times F\left(N=k;\alpha_{3},\beta_{3},E\right)}.$$

In another word, local FDR represents the posterior probability that a drug‐drug‐ADE combination has the background RR (null). As we use PSs to adjust confounding bias, we name the statistic in Equation (17) as adjusted‐FDR for screening adverse DDIs.

### 2.5 Proportional reporting ratio for DDI

Proportional reporting ratio (PRR) is a well‐known method for pharmacovigilance proposed by Evan et al.^30^ PRR is developed for detecting signals of single‐drug‐induced ADE from a SRS database. Specifically, PRR calculates the ratio of ADE reporting rates with and without a drug. Based on the report frequencies of Drug1 and Drug2 (Table 1), PRR_D1_ and PRR_D2_ for Drug1 and Drug2 are defined as:

$${\mathrm{PRR}}_{D1}=\frac{\left(c+g\right)/\left(c+d+g+h\right)}{\left(a+e\right)/\left(a+b+e+f\right)},$$

(18) $${\mathrm{PRR}}_{D2}=\frac{\left(e+g\right)/\left(e+f+g+h\right)}{\left(a+c\right)/\left(a+b+c+d\right)}.$$

For the Drug1‐Drug2‐ADE combination, we define its PRR by considering the Drug1‐Drug2 pair as a “new drug”, based on the drug pair's ADE frequency (Table 1), the PRR_D1D2_ for the Drug1‐Drug2‐ADE combination is:

(19) $${\mathrm{PRR}}_{D1D2}=\frac{g/\left(g+h\right)}{\left(a+c+e\right)/\left(a+b+c+d+e+f\right)}.$$

The lower bound of the 95% confidence interval (CI) for the PRR is defined as:

(20) $${\mathrm{PRR}}_{025}=e^{\ln\;\mathrm{PRR}-1.96\mathrm{SD}},$$

where SD denotes the SD, for Drug1, ${\mathrm{SD}}_{D1}=\sqrt{\frac{1}{c+g}-\frac{1}{c+d+g+h}+\frac{1}{a+e}-\frac{1}{a+b+e+f}}$; similarly, for Drug2 ${\mathrm{SD}}_{D2}=\sqrt{\frac{1}{e+g}-\frac{1}{e+f+g+h}+\frac{1}{a+c}-\frac{1}{a+b+c+d}}$, for Drug1‐Drug2 combination ${\mathrm{SD}}_{D1D2}=\sqrt{\frac{1}{g}-\frac{1}{g+h}+\frac{1}{a+c+e}-\frac{1}{a+b+c+d+e+f}}$.^31^ Let PRR_025 D1_ , PRR_025 D2_ , and PRR_025 D1D2_ to be the values of *PRR*_025_ for Drug1, Drug2, and Drug1‐Drug2 combination, respectively.

Similar to the approach proposed by Almenoff et al^32^, a drug pair with PRR_025 D1D2_ > max(PRR_025 D1_,  PRR_025 D2_) is considered as a signal of DDI.

Comparing to adjusted‐FDR and $\Omega_{025},$ PRR and PRR_025_ are measurements without shrinkage adjustment. Therefore, in this study, they will be served as a reference for the Ω shrinkage and our proposed approach.

## 3 SIMULATION STUDY

We conducted extensive simulation studies to evaluate the performance of our proposed method and compare it with the Ω shrinkage method. In each method, the area under the receiver‐operating characteristic (ROC) curve (AUC) was calculated by using the simulated true positive DDIs and negative controls. Let PS be the PS, *D* be the binary drug exposure status, and *Y* be the binary ADE status. Specifically, the data were simulated as:

1. Simulate drug exposure status by *D* ∼ Bin(1, PS), where PS ∼ Beta(*α*,  *β*).
2. Simulate ADE status for a given drug pair by:

(21) $$\text{Logit}\left[P\left(Y=1\right)\right]=\beta_{0}+\beta_{1}D1+\beta_{2}D2+\beta_{3}D1*D2+\beta_{4}\mathrm{PS}1+\beta_{5}\mathrm{PS}2.$$

In order to evaluate and compare the performance of our proposed method to the Ω shrinkage method, we first define two types of DDIs. One type is DDI with nonpositive individual drug relative ADE risk (DDI‐NPIRR) (eg, *r*_01_ − *r*_00_ ≤ 0 or *r*_10_ − *r*_00_ ≤ 0) and the other is DDI with positive individual drug relative ADE risk (DDI‐PIRR) (eg, both *r*_01_ − *r*_00_ > 0 and *r*_10_ − *r*_00_ > 0).

We simulated data of the following three situations:

- Data contain both DDI‐NPIRR ( *β*_1_ > 0, *β*_2_ < 0, and *β*_3_ > 0) and DDI‐PIRR ( *β*_1_ > 0, *β*_2_ > 0, and *β*_3_ > 0), and without confounding bias ( *β*_4_ = *β*_5_ = 0).
- Data only contain DDI‐NPIRR, and without confounding bias.
- Data contains both DDI‐NPIRR and DDI‐PIRR, and with confounding bias ( *β*_4_ ≠ 0 and *β*_5_ ≠ 0).

For all the three situations, the total number of reports was chosen to be 100 000, and PS was generated from beta distribution such that PS ∼ Beta(1, 6). In each simulation, 10 000 positive DDI signals and 10 000 negative controls were simulated from the uniformly distributed parameters shown in Table 2.

**Table 2:** Parameters setting for the simulation study

	Situation (a)		Situation (b)		Situation (c)	
	DDI signal	Negative control	DDI signal	Negative control	DDI signal	Negative control
*β*_0_	[−6,−5]	[−6,−5]	[−6,−5]	[−6,−5]	[−6,−5]	[−6,−5]
*β*_1_	[−0.8,0.8]	[−0.8,0.8]	[0.1,2]	[−0.8,0.8]	[−0.8,0.8]	[−0.8,0.8]
*β*_2_	[−0.8,0.8]	[−0.8,0.8]	[−2,0.1]	[−0.8,0.8]	[−0.8,0.8]	[−0.8,0.8]
*β*_3_	[0.1,1]	[−1,−0.1]	[0.1,1]	[−1,−0.1]	[0.1,1]	[−1,−0.1]
*β*_4_	0	0	0	0	[−2,1]	[−1,1]
*β*_5_	0	0	0	0	[−2,1]	[−1,1]

Abbreviation: DDI, drug‐drug interaction.

**Figure 2:**
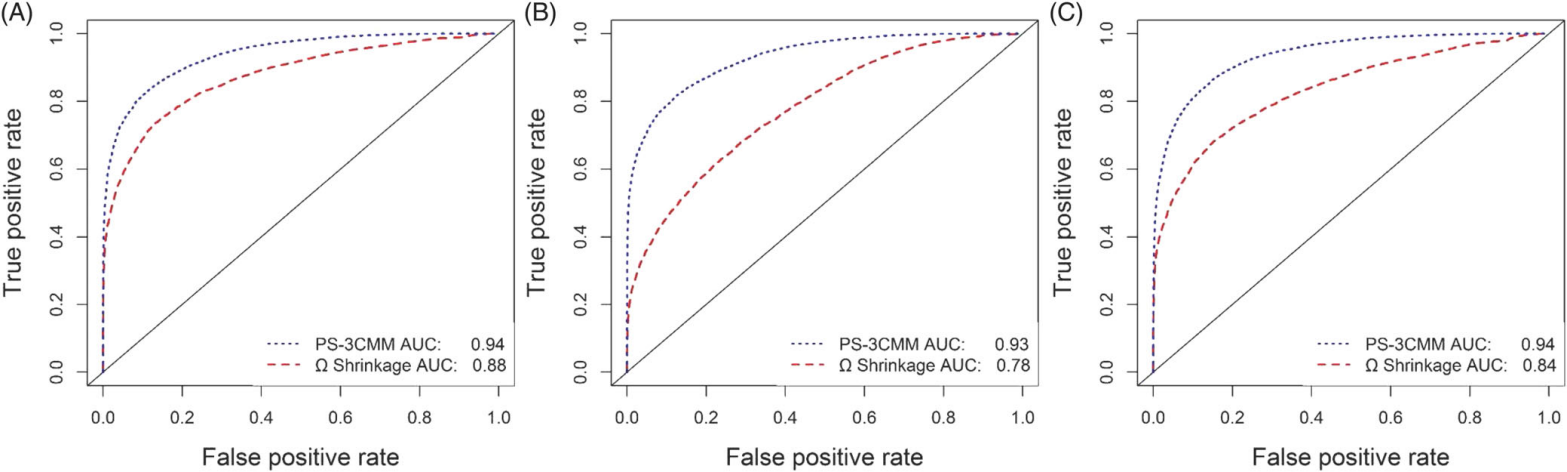
A, Receiver‐operating characteristic (ROC) curves of propensity score‐adjusted three‐component mixture model (PS‐3CMM) and Ω shrinkage without confounding bias for all drug‐drug interactions (DDIs); B, ROC curves of PS‐3CMM and Ω shrinkage without confounding bias for DDI‐nonpositive individual drug relative ADE risk; and C, ROC curves of PS‐3CMM and Ω shrinkage with confounding bias for all DDIs. [Color figure can be viewed at wileyonlinelibrary.com]

The ROC curves and AUC values are shown in Figure 2. In the first simulation, Figure 2A, we display the AUCs in the absence of confounding bias [*β*_4_ = *β*_5_ = 0 ] in Equation (21) for both DDI‐NPIRR and DDI‐PIRR. Although both proposed PS‐3CMM and the Ω shrinkage methods have good performance, PS‐3CMM performs better than Ω shrinkage, AUC = 0.94 vs AUC = 0.88. In the second simulation with only DDI‐NPIRR, PS‐3CMM has even better DDI detection performance than the Ω shrinkage method, AUC 0.93 vs 0.78, respectively. In the third simulation, Figure 2C, when confounding bias is present, [*β*_4_ ≠ 0 and *β*_5_ ≠ 0 ] in Equation (21), the PS‐3CMM's DDI detection performance did not reduce (AUC = 0.94) much from the simulation where there was no confounding. On the other hand, the Ω shrinkage method has obvious decreased performance from 0.88 to 0.84 when confounding bias was presented.

## 4 FAERS ANALYSIS

We demonstrate the utilization of the proposed approach in analyzing the FAERS database including 256 887 drug‐drug‐ADE combinations. In our analysis, 160 PCs, [*K* = 160 ] in Equation (10), were used to calculate the PS and expected report frequency, in which the 160 PCs explained 70% of total variation of all comedications. The estimated parameters for the conditional likelihood in Equation (16) are ${\hat{\alpha }}_{2}={\hat{\beta }}_{2}=2.19$, ${\hat{\alpha }}_{3}=0.22$, ${\hat{\beta }}_{3}=0.04$, and $\hat{\frac{P_{3}}{P_{2}}}=0.08$. These estimates mean that the component 2 (background noise group) had mean RR = 1 with SD = 0.68 and the component 3 (increased risk group) had mean RR = 5.33 with SD = 11.33. For each drug‐drug‐ADE combination, the adjusted‐FDR and $\Omega_{025}$ were calculated for signal detection and comparison. We also calculate the value of PRR_025_ for each drug‐drug‐ADE combination as a reference for the DDI signals detected by PS‐3CMM and Ω shrinkage methods.

### 4.1 Performance comparisons between PS‐3CMM and Ω shrinkage

If both drugs cause an ADE, we define it as the DDI‐PIRR, which yields additive or synergistic risk for the ADE. In terms of reporting rate, DDI‐PIRR has the pattern *r*_11_ > *r*_10_, *r*_01_ > *r*_00_ (Table 1). Alternatively, if only one drug has ADE risk and the other one does not, we define it as DDI‐NPIRR, which increases the ADE risk when two drugs are coprescribed. In terms of reporting rate, DDI‐NPIRR has the pattern *r*_11_ > *r*_10_ > *r*_00_ ≥ *r*_01_ or *r*_11_ > *r*_01_ > *r*_00_ ≥ *r*_10_ .

**Figure 3:**
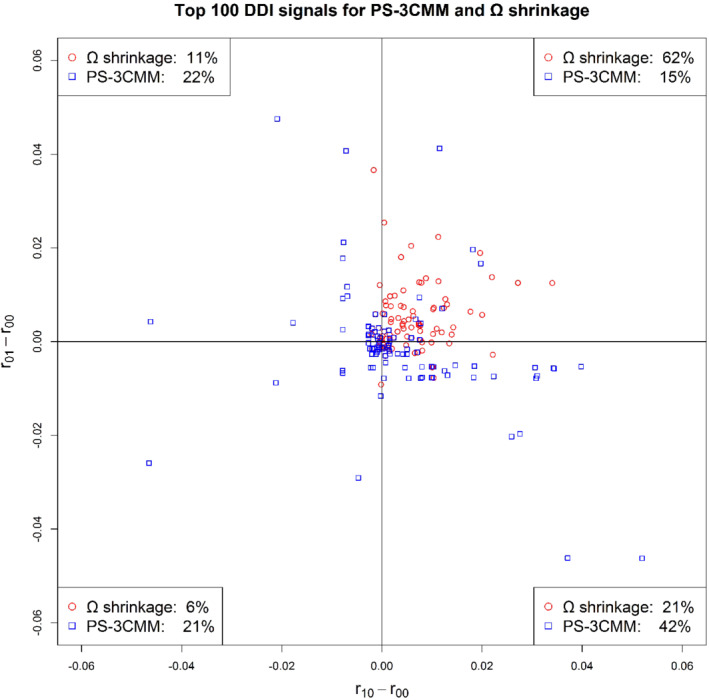
Top‐100 drug‐drug interaction signals for propensity score‐adjusted three‐component mixture model and Ω shrinkage methods. [Color figure can be viewed at wileyonlinelibrary.com]

We compared the top‐100 DDI signals between two methods in Figure 3: PS‐3CMM and Ω shrinkage. The *x*‐axis is the difference of the marginal reporting rate of drug 1 and the reporting rate of no drug (eg, *r*_10_ − *r*_00_ ); and the *y*‐axis is the difference of the marginal reporting rates of drug 2 and the reporting rate of no drug (eg, *r*_01_ − *r*_00_ ). Clearly, top‐100 DDI signals of PS‐3CMM and Ω shrinkage have different patterns. Two lists only have 25 overlapped DDIs (Table 5). Among the Ω shrinkage's top‐100 signals, 62% of them have *r*_10_, *r*_01_ > *r*_00_ . On the other hand, only 15% of the PS‐3CMM's top‐100 signals are DDI‐PIRR. For DDI‐NPIRR ( *r*_00_ ≥ *r*_10_ or *r*_00_ ≥ *r*_01_ ), the percentages are 32% for Ω shrinkage and 64% for PS‐3CMM.

### 4.2 Evaluation of top‐100 DDI signals detected by adjusted‐FDR and $\Omega_{025}$

The drug side‐effect database (SIDER) and DrugBank database were used to evaluate the top‐100 adjusted‐FDR DDI signals (Table S1) and the top‐100 $\Omega_{025}$ DDI signals (Table S2). SIDER collects drug‐ADE association data from drug labels.^33^ DrugBank documented DDIs from a variety of sources.^28^ Our evaluation was conducted manually through searching the drug‐drug‐ADE combinations in SIDER and DrugBank. The results for the adjusted‐FDR DDI signals and the $\Omega_{025}$ DDI signals are summarized in Tables 3 and 4, respectively. As Table 3 shows, 49 out of the top‐100 DDI signals have supporting evidence from DrugBank. Among these 49 DDI signals, seven pairs have both drugs labeled with the ADE; 17 pairs have only one drug labeled with the ADE; and 25 combinations have neither drugs labeled with the ADE. For the remaining 51 DDI signals that are not documented in DrugBank, four have both drugs labeled with the ADE; 20 have only one drug labeled with the ADE; and 27 have neither drug labeled with the ADE. In Table 4, 56 out of the top‐100 DDI signals have supporting evidence from DrugBank. Among these 56 DDI signals, eight pairs have both drugs labeled with the ADE; 25 pairs have only one drug labeled with the ADE; and 23 combinations have neither drugs labeled with the ADE. For the remaining 44 DDI signals that are not documented in DrugBank, one have both drugs labeled with the ADE; 18 have only one drug labeled with the ADE; and 25 have neither drug labeled with the ADE. By using DrugBank as a gold standard to evaluate the top‐100 signals, the proposed method has a positive‐predicted values (PPVs) of 49% and the Ω shrinkage method has a PPV of 56%.

**Table 3:** Evaluation of the top‐100 drug‐drug‐ADE combinations ranked by adjusted‐FDR

	DDI evidence in DrugBank^b^		
Number of drugs have ADE on its label^a^	Yes	No	Total
2	7	4	11
1	17	20	37
0	25	27	52
Total	49	51	100

Abbreviations: ADE, adverse drug event; DDI, drug‐drug interaction; FDR, false discovery rate.

a Drug label information was collected from SIDER.

b DDI evidence was collected from DrugBank.

**Table 4:** Evaluation of the top‐100 drug‐drug‐ADE combinations ranked by $\Omega_{025}$

	DDI evidence in DrugBank^b^		
Number of drugs have ADE on its label^a^	Yes	No	Total
2	8	1	9
1	25	18	43
0	23	25	48
Total	56	44	100

Abbreviations: ADE, adverse drug event; DDI, drug‐drug interaction.

a Drug label information was collected from SIDER.

b DDI evidence was collected from DrugBank.

Using PRR_025_ as a reference, for the top‐100 adjusted‐FDR DDI signals (Table S1), 91 DDI signals have increased PRR_025_ (ie, PRR_025 D1D2_ > max(PRR_025 D1_,  PRR_025 D2_), nine DDI signals have decrease PRR_025_ (ie, PRR_025 D1D2_ < max(PRR_025 D1_,  PRR_025 D2_)). Among these nine DDI signals, four have supporting evidence from DrugBank and two have both drugs labeled with the ADE. In the top‐100 $\Omega_{025}$ DDI signals (Table S2), all of the 100 DDI signals have increased PRR_025_ . Among the top 100 ranking DDI signals detected by adjusted‐FDR and $\Omega_{025}$, there are 25 common DDI signals (Table 5). Among these 25 DDI signals, 14 drug pairs have supporting evidence from DrugBank; only two of the drug pairs have both drugs labeled with ADE and 11 of drug pairs have only one drug labeled with ADE. Moreover, all these 25 common DDI signals have increased PRR_025_ .

**Table 5:** Overlap top‐100 drug‐drug‐ADE combinations

Drug1	Drug2	ADE	−log10(adjusted‐FDR) [Rank]	$\Omega_{025}$ [Rank]	PRR_025 D1_	PRR_025 D2_	PRR_025 D1D2_
BUPROPION a	METHAMPHETAMINE	Neuropathy	35.0380 [9]	3.2731 [84]	2.2115	3.9370	36.1433
BEVACIZUMAB	VALPROIC ACID^a^	Delirium	23.1426 [18]	3.8676 [9]	0.6918	3.2050	66.0302
PEGFILGRASTIM	PHENYTOIN^a^	Delirium	22.7221 [19]	4.9747 [1]	1.9341	2.0910	162.7558
BEVACIZUMAB	PHENYTOIN^a^	Delirium	22.3023 [22]	4.1906 [4]	0.6918	2.0910	49.2345
PEGFILGRASTIM	VALPROIC ACID^a^	Delirium	18.6823 [26]	4.2639 [3]	1.9341	3.2050	106.5403
CARBOPLATIN a	VARENICLINE	Neuropathy	18.4633 [27]	3.6936 [17]	2.2121	0.3180	52.1493
NALOXONE	BUDESONIDE	Neuropathy	17.4483 [28]	3.8090 [13]	0.8758	0.7835	32.8251
PEGFILGRASTIM	BEVACIZUMAB	Delirium	16.6822 [31]	3.4410 [51]	1.9341	0.6918	14.1947
VALPROIC ACID^a^	CARBOPLATIN	Delirium	15.4286 [34]	4.0513 [6]	3.2050	1.5340	89.2514
WARFARIN	TETRACYCLINE	Delirium	14.7344 [38]	3.2537 [93]	1.4521	2.3461	34.1982
METAXALONE	TERIPARATIDE	Delirium	14.0579 [40]	3.2475 [98]	1.2557	0.3933	24.9570
PHENYTOIN	CARBOPLATIN	Delirium	13.3545 [46]	4.3066 [2]	2.0910	1.5340	76.0028
CARVEDILOL	NALOXONE	Neuropathy	13.0354 [49]	3.2530 [94]	1.8283	0.8758	28.1420
METAXALONE	ROSUVASTATIN	Delirium	12.0456 [55]	3.3886 [60]	1.2557	0.5564	24.7431
ROFECOXIB a	LAMOTRIGINE a	Neuropathy	11.8226 [59]	3.5168 [35]	1.9221	1.0212	31.8124
BUPIVACAINE	PREDNISONE a	Delirium	11.2454 [67]	3.4850 [42]	2.0987	0.9646	54.0550
TETRACYCLINE	ESCITALOPRAM a	Delirium	10.9939 [68]	3.3108 [73]	2.3461	1.9282	50.7768
DULOXETINE	ROFECOXIB a	Neuropathy	10.9655 [69]	3.5546 [30]	2.6122	1.9221	45.9861
ROSIGLITAZONE	BIMATOPROST	Neuropathy	10.8976 [71]	3.4377 [52]	1.0349	0.7282	25.5408
GEMCITABINE	DULOXETINE	Delirium	10.6277 [74]	3.3538 [65]	0.8139	1.9728	45.1643
VALDECOXIB a	TELMISARTAN a	Neuropathy	10.6144 [76]	3.4979 [38]	1.1240	0.8844	30.3932
GEMCITABINE	ROFECOXIB	Delirium	10.4108 [79]	3.4159 [56]	0.8139	0.8227	29.1456
BUPIVACAINE	ZOLEDRONATE	Delirium	10.3610 [80]	3.4567 [45]	2.0987	1.1792	58.8019
FILGRASTIM	PHENYTOIN^a^	Delirium	10.2910 [81]	3.8094 [12]	1.9407	2.0910	50.6368
ROFECOXIB	GRANISETRON	Delirium	10.2715 [82]	3.5553 [29]	0.8227	1.4602	42.8081

*Note*: The underlined drug names represent DDI evidence in DrugBank database.

Abbreviations: ADE, adverse drug event; DDI, drug‐drug interaction; FDR, false discovery rate.

a Drugs labeled the ADE in SIDER database.

Except the overlap 25 DDI signals, both our proposed method and the Ω shrinkage method can detect some DDI signals that cannot detected by each other, therefore, the PS‐3CMM is an important complementary to the Ω shrinkage method.

## 5 CONCLUSION AND DISCUSSION

In this article, we propose a new approach, PS‐3CMM to detect signals of DDI from the FAERS database. This method controls false positive rate and adjusts for confounding bias due to comedications. Specifically, this approach first uses the comedications to derive the PSs. These PSs are then utilized to calculate adjusted expected frequencies for drug‐drug‐ADE combinations under the no DDI assumption. The empirical Bayes mixture model is used to characterize the RR between the DDI‐induced ADE frequency and the expected ADE frequency of drug‐drug combination under no DDI effect assumption. This 3CMM estimates adjusted‐FDR for each drug‐drug‐ADE combination.

The PSs are derived from PC analyses of comedication variables. The number of PCs can be determined by the percentage of total variation that explained by the selected PCs. In our illustrative FAERS analysis, the selected PCs capture 70% of variations of comedications. Therefore, we believe these comedication PCs contain sufficient variations among the comedications, and they shall capture a great deal of comorbidity variation among the patients. Our model also includes PS as continuous variable. It avoids the potential sparsity caused by the stratification of the data used in the EBGM and IC.^18^ For instance, in our FAERS database the drug pairs' frequency has a median of 180. For *P* binary variables (eg, age, gender, and comedications), the number of strata is 2^*P*^ . The sparsity appears even for moderate P (eg, stratify by four age groups and gender yield *P* = 8). Unlike the Ω shrinkage method in which the penalize parameter is prespecified, the proposed approach is data‐driven in which the parameters in the prior distribution are estimated from data under the empirical Bayes framework. Furthermore, the adjusted‐FDR provides much needed false positive control for high throughput DDI signals detection. Our simulation studies show the PS‐3CMM has better performance comparing to the Ω shrinkage method especially in detecting DDI‐NPIRR. In addition, the simulation studies also demonstrate that our PS‐3CMM has the advantage of adjusting for confounding bias, which is a common problem in SRS.

The application of PS‐3CMM is demonstrated through the FAERS database. In FAERS analysis, we notice that the PS‐3CMM and the Ω shrinkage prioritized signals differently. PS‐3CMM is more prone to identify DDI‐NPIRR signals. DDI changes the expected pharmacologic or clinical response for an individual drug when two drugs are coadministered.^1^ The incidence of ADE can be significantly increased by: (1) pharmacokinetic (PK) DDI, in which a drug's plasma concentration is increased by coprescribed drug and exceeded the maximum tolerated dose^34^ and (2) PD DDI in which additive or synergistic effects on ADE risk occurs. For DDI‐NPIRR signals, a drug's ADE risk is significantly increased by another drug which has either similar or lower ADE risk compared with background. In another word, DDI‐NPIRR signals are more likely to have a mechanism similar to PK DDI. For instance, the acetaminophen‐methamphetamine‐neuropathy combination and acetaminophen‐neostigmine‐neuropathy combination are highly prioritized by PS‐3CMM but not by the Ω shrinkage method. They are ranked in 11th and 17th by PS‐3CMM, and 915th and 8126th by the Ω shrinkage method (Table S1). In both combinations, the neuropathy risks of methamphetamine and neostigmine are lower than background risks, which imply both of these DDI signals are DDI‐NPIRR signals. Moreover, both drugs are known to have PK drug interaction with acetaminophen according to DrugBank.^35^ For instance, the description of drug interactions for acetaminophen‐methamphetamine combination is “Acetaminophen may decrease the excretion rate of Metamfetamine which could result in a higher serum level”. Thus, these DDI‐NPIRR signals are likely to have a mechanism of PK DDI. Alternatively, DDI‐PIRR signals are more likely to have a mechanism similar to PD DDI. For DDI‐PIRR signals, both individual drugs have higher than background risks, and the drug combinations have much increased risks. For instance, the cetirizine‐levodopa‐neuropathy combination and the ibuprofen‐tinzaparin‐neuropathy combination are highly prioritized by Ω shrinkage but not by the PS‐3CMM. They are ranked in 32th and 34th by the Ω shrinkage method and 5716th and 19226th by PS‐3CMM (Table S2). Both of these two DDI signals are DDI‐PIRR signals. These two‐drug combinations are known to have increased ADE risk according to DrugBank with no clear evidence of PK drug interaction.^35^ In conclusion, DDI‐NPIRR signals are more likely to have a mechanism similar to PK DDI and DDI‐PIRR signals are more likely to have a mechanism of PD DDI. The difference of the DDI signals pattern is mainly caused by the different baseline risk assumptions, and the Ω shrinkage method also restricts the ADE risk caused by single drug should be higher than its background risk, but the PS‐3CMM do not has such restriction. Among the top‐100 signals detected by PS‐3CMM, 49 drug pairs have documented DDI evidences from DrugBank and 24 drug pairs have at least one drug with the ADE in its drug label. At the meantime, 75% of top 100 PS‐3CMM DDI signals are not ranked top 100 by the Ω shrinkage method. These results suggest that PS‐3CMM is a different and complementary approach to the Ω shrinkage approach, as well as the PRR approach.

As we described earlier in Section 2.4.1, instead of using PS‐adjusted expected DDI‐ADE frequency under the no DDI effect assumption, unadjusted expected DDI‐ADE frequency can be computed in Equation (9). Although PS‐adjusted analysis is clearly supreme to the unadjusted analysis in FAERS data analysis, there are other situations that unadjusted analysis is probably better. For example, if the pharmaco‐epidemiological study is designed from a longitudinal cohort, such as the electronic health record (EHR) dataset, the confounding variables, such as demographics, comedications, or comorbidity can be matched and adjusted through nested case‐control study design.^36^, ^37^ In this case, in analyzing DDI‐ADE associations, the unadjusted analysis is a proper choice. Because it does not need to adjust for confounding variables, it shall have better power comparing to the adjusted analysis. Additionally, in real application, either the full model (Equation 13) or the conditional model (Equation 15) can be used for signal detection. However, the conditional model is more efficient when the primary interest is to detect drug‐drug‐ADE combinations with increased risk.

In this article, we illustrate the utilization of our proposed method by using the FAERS database which is a preeminent SRSs. Here, we would like to point that the proposed method can be utilized to other SRS databases. Nowadays, the importance of pharmacovigilance through SRS is internationally recognized. For instance, all European Union countries were obliged to establish patient/consumer reporting within their SRSs since 2012.^38^ China and Japan have well‐established SRS as well.^39^, ^40^ Additionally, the WHOs SRS (VigiBase) contains over 20 million reports which are coded by MedDRA and WHO Drug Dictionaries.^41^ A detailed review of SRS database can be found in Huang et al^42^ These SRS databases share similar informatics structure (eg, MedDRA), which facilitates the utilization of the proposed method. For instance, the Ω Shrinkage method has been applied to both VigiBase and the China's SRS database,^16^, ^43^, ^44^, ^45^ in which the frequencies in Table 1 are able to be obtained. Thus, PS‐3CMM can be straightforwardly implemented to the other SRS databases, which share similar informatics structure with FAERS. As for illustration, we only choose four ADEs in our FAERS analysis. However, the framework of PS‐3CMM has no limitation with respect to the number of ADEs. We would also like to point out that the study can be initiated by either ADE(s) or drug combination(s). The current study is initiated by ADEs, as our motivation is to detect novel drug interaction signals. Alternatively, a study may be initiated with drug combinations with evidence of DDI. Such a study is more powerful to detect ADE signals and it can be used as a validation for pharmacology study.

One limitation of the proposed approach is to handle highly correlated drugs. It is a major challenge for all of the current DDI detection research. These highly correlated drugs (eg, a drug always coadministered with another) are due to increasing practice of polypharmacy. Another limitation is the proposed approach does not incorporate the drugs' duration and dosage information which are important factors for DDI, as those informations are not well captured by most SRS databases. Alternatively, EHRs databases contain detailed temporal information with respect to patients' pharmacy prescriptions, as well as the dosages. The associations between drugs and ADEs in EHR can be investigated under elegant epidemiology designs. For instance, nested case‐control design with a 30‐day drug exposure can be used to investigate acute ADEs, as well as the case‐crossover design.^46^ Hennessy et al provides detailed guidance for investigating DDI from EHR databases.^47^ We would like to point out that our proposed method has the potential to analyze EHR data with proper epidemiology designs. For instance, instantaneous exposure before the ADE (ie, duration information) can be captured by the drug exposure window, and the analysis can be stratified or adjusted by dosage information. We believe the detection of DDI signals from EHR databases can be an important direction to further expand our model in order to characterize drugs' duration and dosage information.

## Supporting information


**Data S1** Supporting Information

## Data Availability

Data sharing is not applicable to this article as no new data were created or analyzed in this study.
