# Supplementary material for: Propensity score‐adjusted three‐component mixture model for drug‐drug interaction data mining in FDA Adverse Event Reporting System
Source: Stat Med. 2019 Dec 27;39(7):996–1010. doi: 10.1002/sim.8457 (PMC9292662; doi:10.1002/sim.8457)
Supplement: Supplementary file 1 — Data S1 Supporting Information [file SIM-39-996-s001.docx]

**Table S1.** The top-100 DDI signals detected by the adjusted-FDR. The underlined name drug represents the drug-drug pair is documented to have drug-drug interaction in DrugBank database and the drug name with # represents the drug labeled with the ADE in SIDER database.

| drug1 | drug2 | ADE | r_00_ | r_10_ | r_01_ | r_11_ | N | -log10(adjusted-FDR) [Rank] | Ω025 [Rank] | -log10[FDR] [Rank] | $\mathrm{PRR}_{025 D1}$ | $\mathrm{PRR}_{025 D2}$ | $\mathrm{PRR}_{025 D1D2}$ |
| --- | --- | --- | --- | --- | --- | --- | --- | --- | --- | --- | --- | --- | --- |
| RITONAVIR^#^ | LOPINAVIR^#^ | Neuropathy | 0.0078 | 0.0155 | 0.0000 | 0.0172 | 205 | 269.0991 [1] | -0.0619 [80115] | 195.9469 [1] | 1.9010 | 1.8927 | 1.9154 |
| NORGESTIMATE | ETHINYL ESTRADIOL | Myopathy | 0.0465 | 0.0000 | 0.0206 | 0.0375 | 128 | 131.9872 [2] | -0.5706 [115911] | 117.6716 [3] | 0.6772 | 0.4429 | 0.6838 |
| NORGESTIMATE | ETHINYL ESTRADIOL | Skin pigmentation disorder | 0.0212 | 0.0000 | 0.0124 | 0.0182 | 62 | 62.7545 [3] | -0.6021 [118083] | 52.5622 [5] | 0.6660 | 0.5579 | 0.6725 |
| TRETINOIN^#^ | NORGESTIMATE | Myopathy | 0.0463 | 0.0416 | 0.0172 | 0.1061 | 82 | 62.4389 [4] | 0.8555 [27637] | 3.0434 [2010] | 0.8890 | 0.6772 | 1.8689 |
| ATOVAQUONE^#^ | PROGUANIL | Myopathy | 0.0463 | 0.0982 | 0.0000 | 0.0381 | 60 | 53.2321 [5] | -1.7485 [177245] | 44.6498 [6] | 0.9507 | 0.6315 | 0.6420 |
| RITONAVIR | LOPINAVIR | Delirium | 0.0027 | 0.0014 | 0.0000 | 0.0028 | 34 | 52.2472 [6] | -0.4525 [107994] | 33.6021 [7] | 0.5875 | 0.7447 | 0.7537 |
| CLAVULANATE | AMOXICILLIN | Neuropathy | 0.0077 | 0.0000 | 0.0288 | 0.0312 | 481 | 44.1421 [7] | -0.0166 [76952] | 90.8665 [4] | 3.6854 | 3.6735 | 3.6868 |
| CLAVULANATE | AMOXICILLIN | Delirium | 0.0027 | 0.0000 | 0.0040 | 0.0036 | 56 | 41.5376 [8] | -0.5502 [114608] | 30.3969 [8] | 1.0383 | 1.2033 | 1.0387 |
| BUPROPION^#^ | METHAMPHETAMINE | Neuropathy | 0.0077 | 0.0178 | 0.0000 | 0.3898 | 23 | 35.0381 [9] | 3.2731 [84] | 22.2097 [10] | 2.2115 | 3.9370 | 36.1433 |
| GABAPENTIN^#^ | METHAMPHETAMINE | Neuropathy | 0.0073 | 0.0383 | 0.0000 | 0.4423 | 23 | 34.4763 [10] | 2.5876 [758] | 22.2013 [11] | 5.0904 | 3.9370 | 41.5881 |
| ACETAMINOPHEN | METHAMPHETAMINE | Neuropathy | 0.0072 | 0.0203 | 0.0000 | 0.2190 | 23 | 34.0458 [11] | 2.5115 [915] | 22.2007 [12] | 2.7451 | 3.9370 | 19.4755 |
| ACETAMINOPHEN | PHENAZOPYRIDINE | Neuropathy | 0.0072 | 0.0203 | 0.0000 | 0.2091 | 23 | 32.4510 [12] | 2.4569 [1069] | 22.1694 [13] | 2.7451 | 7.3517 | 18.5476 |
| GADOTERIDOL | GADOBENATE DIMEGLUMINE | Neuropathy | 0.0078 | 0.0000 | 0.0011 | 0.0112 | 19 | 28.3716 [13] | -0.2205 [91613] | 17.9136 [17] | 0.7219 | 0.5037 | 0.9152 |
| GADOTERIDOL | GADOPENTETATE DIMEGLUMINE | Neuropathy | 0.0078 | 0.0000 | 0.0104 | 0.0108 | 19 | 27.8327 [14] | -0.6667 [122332] | 17.8182 [19] | 0.7219 | 1.0868 | 0.8817 |
| GADODIAMIDE^#^ | GADOTERIDOL | Neuropathy | 0.0078 | 0.0132 | 0.0000 | 0.0108 | 19 | 27.6253 [15] | -0.9976 [142251] | 17.7878 [20] | 1.1938 | 0.7219 | 0.8833 |
| GADOVERSETAMIDE | GADOTERIDOL | Neuropathy | 0.0078 | 0.0386 | 0.0000 | 0.0114 | 19 | 26.7645 [16] | -2.4544 [200087] | 17.5406 [21] | 2.0588 | 0.7219 | 0.9306 |
| ACETAMINOPHEN | NEOSTIGMINE | Neuropathy | 0.0072 | 0.0203 | 0.0000 | 0.1212 | 16 | 23.2076 [17] | 1.5831 [8126] | 14.7144 [31] | 2.7451 | 2.8219 | 9.7673 |
| BEVACIZUMAB | VALPROIC ACID^#^ | Delirium | 0.0026 | 0.0013 | 0.0085 | 0.2346 | 38 | 23.1427 [18] | 3.8676 [9] | 18.1864 [16] | 0.6918 | 3.2050 | 66.0302 |
| PEGFILGRASTIM | PHENYTOIN^#^ | Delirium | 0.0027 | 0.0031 | 0.0048 | 0.5429 | 38 | 22.7212 [19] | 4.9747 [1] | 23.3188 [9] | 1.9341 | 2.0910 | 162.7558 |
| FENTANYL^#^ | NEOSTIGMINE | Neuropathy | 0.0077 | 0.0176 | 0.0000 | 0.0637 | 16 | 22.6882 [20] | 0.9528 [23990] | 14.6676 [34] | 2.1887 | 2.8219 | 5.0611 |
| PROPOFOL^#^ | NEOSTIGMINE | Neuropathy | 0.0078 | 0.0082 | 0.0000 | 0.0611 | 16 | 22.3969 [21] | 1.8455 [4666] | 14.7011 [32] | 0.9961 | 2.8219 | 4.8453 |
| BEVACIZUMAB | PHENYTOIN^#^ | Delirium | 0.0027 | 0.0013 | 0.0048 | 0.1754 | 40 | 22.3019 [22] | 4.1906 [4] | 22.1561 [14] | 0.6918 | 2.0910 | 49.2345 |
| FUROSEMIDE^#^ | PROCAINAMIDE^#^ | Skin pigmentation disorder | 0.0203 | 0.0462 | 0.0000 | 0.2258 | 14 | 19.7773 [23] | 1.2551 [14854] | 12.5768 [48] | 2.2244 | 2.0540 | 6.7493 |
| AMSACRINE^#^ | CYTARABINE^#^ | Myopathy | 0.0462 | 0.0000 | 0.0505 | 0.1163 | 20 | 19.5528 [24] | 0.4559 [46607] | 15.9586 [26] | 1.6258 | 1.0339 | 1.6647 |
| ZOLEDRONATE | VARENICLINE | Neuropathy | 0.0074 | 0.0417 | 0.0017 | 0.3700 | 84 | 18.8508 [25] | 2.7603 [438] | 21.3925 [15] | 5.6373 | 0.3180 | 39.9092 |
| PEGFILGRASTIM | VALPROIC ACID^#^ | Delirium | 0.0026 | 0.0031 | 0.0085 | 0.3689 | 38 | 18.6819 [26] | 4.2639 [3] | 14.4330 [38] | 1.9341 | 3.2050 | 106.5403 |
| CARBOPLATIN^#^ | VARENICLINE | Neuropathy | 0.0079 | 0.0178 | 0.0025 | 0.5294 | 27 | 18.4634 [27] | 3.6936 [17] | 17.4828 [22] | 2.2121 | 0.3180 | 52.1493 |
| NALOXONE | BUDESONIDE | Neuropathy | 0.0079 | 0.0059 | 0.0064 | 0.3621 | 21 | 17.4486 [28] | 3.8090 [13] | 13.1979 [45] | 0.8758 | 0.7835 | 32.8251 |
| LENALIDOMIDE^#^ | CYCLOPHOSPHAMIDE^#^ | Neuropathy | 0.0074 | 0.0272 | 0.0241 | 0.1529 | 142 | 17.3788 [29] | 1.5641 [8445] | 0.0543 [67518] | 3.7127 | 3.3156 | 16.8172 |
| LEVOTHYROXINE | PILOCARPINE | Neuropathy | 0.0076 | 0.0143 | 0.0124 | 0.0697 | 17 | 17.3410 [30] | 0.9991 [22363] | 0.1967 [25284] | 1.8089 | 1.9946 | 5.6189 |
| PEGFILGRASTIM | BEVACIZUMAB | Delirium | 0.0027 | 0.0029 | 0.0012 | 0.0511 | 43 | 16.6819 [31] | 3.4410 [51] | 16.2976 [25] | 1.9341 | 0.6918 | 14.1947 |
| ACETAMINOPHEN^#^ | RIMANTADINE | Skin pigmentation disorder | 0.0197 | 0.0473 | 0.0000 | 0.1667 | 12 | 15.5670 [32] | 0.7489 [32089] | 10.4045 [77] | 2.3506 | 2.4178 | 4.7121 |
| ROFECOXIB^#^ | PILOCARPINE | Neuropathy | 0.0077 | 0.0155 | 0.0116 | 0.2254 | 16 | 15.4330 [33] | 2.3489 [1419] | 2.7817 [2345] | 1.9221 | 1.9946 | 18.6760 |
| VALPROIC ACID^#^ | CARBOPLATIN | Delirium | 0.0026 | 0.0085 | 0.0034 | 0.3136 | 37 | 15.4283 [34] | 4.0513 [6] | 11.8894 [53] | 3.2050 | 1.5340 | 89.2514 |
| PEGINTERFERON ALFA-2B | BOCEPREVIR | Delirium | 0.0027 | 0.0077 | 0.0000 | 0.0185 | 11 | 15.1244 [35] | 0.2112 [61357] | 9.4609 [104] | 2.5181 | 1.2896 | 3.8252 |
| WARFARIN | SILVER SULFADIAZINE | Neuropathy | 0.0076 | 0.0191 | 0.0488 | 0.2167 | 13 | 15.0123 [36] | 0.8422 [28192] | 0.0545 [67277] | 2.4178 | 6.4026 | 17.0817 |
| AMPHOTERICIN B^#^ | FLUCYTOSINE^#^ | Myopathy | 0.0462 | 0.0833 | 0.0000 | 0.0656 | 12 | 14.7423 [37] | -1.2628 [156383] | 9.9788 [91] | 1.6591 | 0.6306 | 0.8202 |
| WARFARIN | TETRACYCLINE | Delirium | 0.0027 | 0.0041 | 0.0007 | 0.1471 | 15 | 14.7352 [38] | 3.2537 [93] | 11.1637 [61] | 1.4521 | 2.3461 | 34.1982 |
| EPTIFIBATIDE^#^ | LISINOPRIL^#^ | Neuropathy | 0.0076 | 0.0006 | 0.0193 | 0.1329 | 21 | 14.2351 [39] | 1.9174 [3972] | 13.6576 [43] | 1.0394 | 2.4546 | 11.3860 |
| METAXALONE | TERIPARATIDE | Delirium | 0.0027 | 0.0022 | 0.0011 | 0.1102 | 14 | 14.0580 [40] | 3.2475 [98] | 10.2549 [82] | 1.2557 | 0.3933 | 24.9570 |
| METFORMIN | RALTEGRAVIR^#^ | Delirium | 0.0027 | 0.0018 | 0.0007 | 0.1204 | 13 | 13.8210 [41] | 3.1978 [116] | 10.3706 [79] | 0.6320 | 0.6957 | 26.8241 |
| ETONOGESTREL | ETHINYL ESTRADIOL | Delirium | 0.0027 | 0.0007 | 0.0000 | 0.0013 | 11 | 13.5686 [42] | -1.9839 [185856] | 8.2644 [169] | 0.2518 | 0.0594 | 0.2717 |
| CLIDINIUM | ACETYLSALICYLIC ACID | Delirium | 0.0026 | 0.0000 | 0.0042 | 0.0562 | 10 | 13.5058 [43] | 2.0459 [2987] | 8.4260 [159] | 1.8110 | 1.5198 | 11.4129 |
| CYCLOPHOSPHAMIDE^#^ | VARENICLINE | Neuropathy | 0.0077 | 0.0262 | 0.0025 | 0.4028 | 29 | 13.4881 [44] | 3.0548 [176] | 13.9872 [39] | 3.3156 | 0.3180 | 38.7950 |
| DEXAMETHASONE^#^ | VARENICLINE | Neuropathy | 0.0073 | 0.0471 | 0.0020 | 0.3681 | 67 | 13.3990 [45] | 2.5294 [877] | 15.6737 [27] | 6.4358 | 0.3180 | 38.8744 |
| PHENYTOIN^#^ | CARBOPLATIN | Delirium | 0.0027 | 0.0050 | 0.0035 | 0.2713 | 35 | 13.3546 [46] | 4.3066 [2] | 14.7447 [30] | 2.0910 | 1.5340 | 76.0028 |
| CARBIDOPA | LEVODOPA | Skin pigmentation disorder | 0.0211 | 0.0033 | 0.0251 | 0.0198 | 202 | 13.3107 [47] | -0.5463 [114338] | 1.6697 [4799] | 0.8003 | 0.8889 | 0.8195 |
| METHYLPREDNISOLONE^#^ | VARENICLINE | Neuropathy | 0.0079 | 0.0158 | 0.0025 | 0.2013 | 30 | 13.1605 [48] | 2.8515 [331] | 13.1409 [46] | 1.9771 | 0.3180 | 18.6608 |
| CARVEDILOL | NALOXONE | Neuropathy | 0.0078 | 0.0148 | 0.0055 | 0.3077 | 24 | 13.0353 [49] | 3.2530 [94] | 9.4001 [107] | 1.8283 | 0.8758 | 28.1420 |
| EPOETIN ALFA | VARENICLINE | Neuropathy | 0.0078 | 0.0384 | 0.0023 | 0.4259 | 46 | 12.9830 [50] | 2.8768 [311] | 16.3010 [24] | 4.9186 | 0.3180 | 43.6825 |
| CHLORDIAZEPOXIDE | CLIDINIUM | Delirium | 0.0027 | 0.0069 | 0.0000 | 0.0095 | 10 | 12.7282 [51] | -0.5959 [117623] | 8.2464 [170] | 1.9347 | 1.8110 | 1.9061 |
| PROCHLORPERAZINE | VARENICLINE | Neuropathy | 0.0078 | 0.0422 | 0.0021 | 0.2913 | 60 | 12.6536 [52] | 2.3300 [1494] | 13.7670 [42] | 5.4900 | 0.3180 | 30.0616 |
| RITUXIMAB | CONJUGATED ESTROGENS | Delirium | 0.0027 | 0.0010 | 0.0012 | 0.1833 | 11 | 12.4225 [53] | 3.1379 [140] | 8.9208 [128] | 0.3834 | 0.3994 | 39.8718 |
| ZOLPIDEM^#^ | LENALIDOMIDE^#^ | Neuropathy | 0.0073 | 0.0255 | 0.0270 | 0.1413 | 153 | 12.0675 [54] | 1.4204 [11132] | 0.0403 [94075] | 3.4816 | 3.7127 | 15.6188 |
| METAXALONE | ROSUVASTATIN | Delirium | 0.0027 | 0.0017 | 0.0016 | 0.1060 | 16 | 12.0458 [55] | 3.3886 [60] | 11.3002 [60] | 1.2557 | 0.5564 | 24.7431 |
| LOTEPREDNOL | ACETYLSALICYLIC ACID | Delirium | 0.0026 | 0.0000 | 0.0042 | 0.0928 | 9 | 11.8633 [56] | 2.2968 [1616] | 7.3958 [246] | 2.5690 | 1.5198 | 18.4719 |
| PYRIDOXAL PHOSPHATE | ISONIAZID | Delirium | 0.0027 | 0.0000 | 0.0060 | 0.0148 | 9 | 11.8539 [57] | 0.1137 [67809] | 7.3925 [248] | 1.0791 | 1.8821 | 2.8752 |
| ACETAMINOPHEN | NITROPRUSSIDE | Neuropathy | 0.0072 | 0.0203 | 0.0000 | 0.0833 | 9 | 11.8386 [58] | 0.7246 [33159] | 7.3904 [249] | 2.7451 | 1.2678 | 5.6859 |
| ROFECOXIB^#^ | LAMOTRIGINE^#^ | Neuropathy | 0.0077 | 0.0152 | 0.0080 | 0.3271 | 35 | 11.8239 [59] | 3.5168 [35] | 9.0391 [122] | 1.9221 | 1.0212 | 31.8124 |
| SIMVASTATIN^#^ | PAPAVERINE | Neuropathy | 0.0076 | 0.0156 | 0.0000 | 0.0947 | 9 | 11.8041 [60] | 1.1652 [17310] | 7.3851 [251] | 1.9684 | 1.5672 | 6.4893 |
| OXYCODONE | METHYLNALTREXONE | Neuropathy | 0.0074 | 0.0297 | 0.0000 | 0.1875 | 12 | 11.7055 [61] | 1.4494 [10516] | 10.4895 [75] | 3.8576 | 3.4141 | 14.3610 |
| AMSACRINE^#^ | CYTARABINE^#^ | Skin pigmentation disorder | 0.0209 | 0.0000 | 0.0685 | 0.0988 | 17 | 11.5638 [62] | -0.2555 [94180] | 9.8601 [95] | 2.9134 | 3.0936 | 2.9829 |
| INSULIN GLARGINE^#^ | MILRINONE | Neuropathy | 0.0077 | 0.0180 | 0.0023 | 0.2879 | 19 | 11.5482 [63] | 2.8059 [381] | 10.6144 [71] | 2.2316 | 1.3662 | 25.1287 |
| NORGESTIMATE | ETHINYL ESTRADIOL | Neuropathy | 0.0079 | 0.0000 | 0.0017 | 0.0029 | 10 | 11.4921 [64] | -2.4181 [199131] | 8.0675 [183] | 0.1992 | 0.1848 | 0.2011 |
| METHADONE | EXENATIDE^#^ | Neuropathy | 0.0078 | 0.0225 | 0.0028 | 0.3134 | 21 | 11.4157 [65] | 2.7396 [476] | 10.0061 [90] | 2.7544 | 0.3487 | 28.0549 |
| ZOLEDRONATE | SUMATRIPTAN^#^ | Skin pigmentation disorder | 0.0211 | 0.0209 | 0.0095 | 0.3295 | 29 | 11.3316 [66] | 3.0710 [165] | 11.8894 [54] | 0.9568 | 0.4761 | 11.5942 |
| BUPIVACAINE | PREDNISONE^#^ | Delirium | 0.0027 | 0.0042 | 0.0028 | 0.2273 | 15 | 11.2457 [67] | 3.4850 [42] | 8.6271 [146] | 2.0987 | 0.9646 | 54.0550 |
| TETRACYCLINE | ESCITALOPRAM^#^ | Delirium | 0.0027 | 0.0007 | 0.0055 | 0.2143 | 15 | 10.9957 [68] | 3.3108 [73] | 11.3799 [59] | 2.3461 | 1.9282 | 50.7768 |
| DULOXETINE | ROFECOXIB^#^ | Neuropathy | 0.0076 | 0.0196 | 0.0146 | 0.4229 | 85 | 10.9666 [69] | 3.5546 [30] | 6.4921 [348] | 2.6122 | 1.9221 | 45.9861 |
| METOLAZONE | TERIPARATIDE | Delirium | 0.0027 | 0.0077 | 0.0011 | 0.1842 | 14 | 10.9586 [70] | 2.8848 [299] | 8.4647 [155] | 3.0039 | 0.3933 | 42.5887 |
| ROSIGLITAZONE | BIMATOPROST | Neuropathy | 0.0078 | 0.0085 | 0.0048 | 0.2951 | 18 | 10.8962 [71] | 3.4377 [52] | 10.4045 [78] | 1.0349 | 0.7282 | 25.5408 |
| VINORELBINE | NAPROXEN | Delirium | 0.0027 | 0.0026 | 0.0015 | 0.0993 | 14 | 10.8268 [72] | 3.1852 [122] | 8.7959 [135] | 1.2879 | 0.5323 | 22.4109 |
| TIOTROPIUM | DESVENLAFAXINE | Neuropathy | 0.0079 | 0.0056 | 0.0023 | 0.1585 | 13 | 10.6517 [73] | 2.6681 [601] | 8.9706 [126] | 0.6675 | 0.3141 | 12.2815 |
| GEMCITABINE | DULOXETINE | Delirium | 0.0027 | 0.0020 | 0.0056 | 0.1868 | 17 | 10.6271 [74] | 3.3538 [65] | 9.0625 [119] | 0.8139 | 1.9728 | 45.1643 |
| CYCLOPHOSPHAMIDE | BUSULFAN^#^ | Delirium | 0.0027 | 0.0017 | 0.0004 | 0.0119 | 21 | 10.6198 [75] | 1.3460 [12741] | 10.6946 [69] | 0.6421 | 1.3029 | 2.8898 |
| VALDECOXIB^#^ | TELMISARTAN^#^ | Neuropathy | 0.0078 | 0.0092 | 0.0071 | 0.3462 | 18 | 10.6144 [76] | 3.4979 [38] | 9.1512 [114] | 1.1240 | 0.8844 | 30.3932 |
| RAMIPRIL | QUININE^#^ | Delirium | 0.0027 | 0.0039 | 0.0019 | 0.0792 | 24 | 10.5850 [77] | 3.2208 [102] | 9.1367 [115] | 1.4451 | 1.8838 | 20.0368 |
| TRAMADOL | NORETHINDRONE | Neuropathy | 0.0077 | 0.0202 | 0.0015 | 0.1948 | 15 | 10.5229 [78] | 2.0927 [2699] | 8.5100 [152] | 2.4945 | 0.3780 | 15.7795 |
| GEMCITABINE | ROFECOXIB | Delirium | 0.0027 | 0.0021 | 0.0024 | 0.1261 | 15 | 10.4112 [79] | 3.4159 [56] | 9.4559 [106] | 0.8139 | 0.8227 | 29.1456 |
| BUPIVACAINE | ZOLEDRONATE | Delirium | 0.0027 | 0.0042 | 0.0034 | 0.2459 | 15 | 10.3605 [80] | 3.4567 [45] | 8.2343 [171] | 2.0987 | 1.1792 | 58.8019 |
| FILGRASTIM | PHENYTOIN^#^ | Delirium | 0.0027 | 0.0040 | 0.0051 | 0.1854 | 33 | 10.2907 [81] | 3.8094 [12] | 9.9508 [92] | 1.9407 | 2.0910 | 50.6368 |
| ROFECOXIB | GRANISETRON | Delirium | 0.0027 | 0.0024 | 0.0035 | 0.1798 | 16 | 10.2716 [82] | 3.5553 [29] | 9.5544 [100] | 0.8227 | 1.4602 | 42.8081 |
| PAROXETINE^#^ | PILOCARPINE | Neuropathy | 0.0077 | 0.0152 | 0.0171 | 0.1509 | 8 | 10.2381 [83] | 1.0735 [19972] | 0.4007 [15873] | 1.8652 | 1.9946 | 10.1657 |
| METOCLOPRAMIDE | PANCURONIUM | Neuropathy | 0.0076 | 0.0259 | 0.0000 | 0.1481 | 8 | 10.2336 [84] | 0.9924 [22635] | 6.3726 [361] | 3.2279 | 0.4985 | 9.9670 |
| OPRELVEKIN | FILGRASTIM | Delirium | 0.0027 | 0.0000 | 0.0059 | 0.1250 | 8 | 10.2240 [85] | 2.1111 [2581] | 6.3655 [367] | 8.2797 | 1.9407 | 24.2473 |
| ACETYLSALICYLIC ACID | PROCAINAMIDE | Delirium | 0.0026 | 0.0042 | 0.0000 | 0.1569 | 8 | 10.2233 [86] | 2.4056 [1216] | 6.3716 [362] | 1.5198 | 7.7197 | 30.7925 |
| DEXRAZOXANE | DOXORUBICIN^#^ | Delirium | 0.0027 | 0.0000 | 0.0024 | 0.0491 | 8 | 10.2218 [87] | 2.0089 [3273] | 6.3655 [368] | 5.2952 | 0.7920 | 9.2617 |
| FUROSEMIDE | PROCAINAMIDE | Delirium | 0.0026 | 0.0057 | 0.0000 | 0.1290 | 8 | 10.2182 [88] | 2.1472 [2365] | 6.3706 [363] | 2.0642 | 7.7197 | 25.0669 |
| ESMOLOL^#^ | CEFAZOLIN | Neuropathy | 0.0078 | 0.0000 | 0.0256 | 0.0899 | 8 | 10.1618 [89] | 0.4465 [47155] | 6.3575 [373] | 1.4047 | 2.9013 | 5.9187 |
| FORMOTEROL | DESVENLAFAXINE | Neuropathy | 0.0079 | 0.0061 | 0.0023 | 0.1781 | 13 | 10.1427 [90] | 2.7620 [435] | 9.0458 [121] | 0.7205 | 0.3141 | 13.8763 |
| INFLIXIMAB | CEVIMELINE | Neuropathy | 0.0079 | 0.0070 | 0.0090 | 0.1379 | 8 | 10.1373 [91] | 1.8882 [4262] | 2.7113 [2471] | 0.8258 | 1.4660 | 9.2442 |
| ESMOLOL^#^ | MIDAZOLAM^#^ | Neuropathy | 0.0078 | 0.0000 | 0.0170 | 0.0650 | 8 | 10.1361 [92] | 0.5453 [41854] | 6.3585 [371] | 1.4047 | 1.9415 | 4.2444 |
| FILGRASTIM | BEVACIZUMAB | Delirium | 0.0027 | 0.0039 | 0.0014 | 0.0608 | 36 | 10.0429 [93] | 3.1773 [124] | 12.2118 [50] | 1.9407 | 0.6918 | 16.4697 |
| NORETHINDRONE | ESTRADIOL | Delirium | 0.0027 | 0.0003 | 0.0012 | 0.0078 | 12 | 9.8894 [94] | 0.4856 [45000] | 7.2882 [260] | 0.5728 | 0.4356 | 1.6375 |
| CAPECITABINE | GLUCOSAMINE | Delirium | 0.0027 | 0.0021 | 0.0019 | 0.1930 | 11 | 9.8447 [95] | 3.1599 [132] | 7.9626 [192] | 0.7201 | 0.8962 | 42.1033 |
| CYCLOPENTOLATE | DEXAMETHASONE^#^ | Neuropathy | 0.0072 | 0.0000 | 0.0479 | 0.1026 | 8 | 9.8182 [96] | -0.1641 [87521] | 6.3439 [377] | 1.4243 | 6.4358 | 6.7847 |
| LAMOTRIGINE^#^ | MEDROXYPROGESTERONE | Neuropathy | 0.0079 | 0.0086 | 0.0034 | 0.2182 | 12 | 9.8097 [97] | 2.7549 [451] | 7.2798 [261] | 1.0212 | 0.3972 | 16.8742 |
| CONJUGATED ESTROGENS | ZOLEDRONATE | Delirium | 0.0027 | 0.0010 | 0.0032 | 0.0367 | 26 | 9.7878 [98] | 2.6413 [657] | 10.5243 [73] | 0.3994 | 1.1792 | 9.3390 |
| EPTIFIBATIDE^#^ | METOPROLOL | Neuropathy | 0.0076 | 0.0007 | 0.0173 | 0.0648 | 21 | 9.6655 [99] | 1.1347 [18180] | 10.2958 [81] | 1.0394 | 2.1934 | 5.4677 |
| CLOPIDOGREL^#^ | VECURONIUM | Neuropathy | 0.0078 | 0.0124 | 0.0022 | 0.2143 | 15 | 9.6635 [100] | 2.6835 [573] | 9.0711 [118] | 1.5152 | 0.8270 | 17.4536 |

**Table S2.** The top-100 DDI signals detected by the Ω025. The underlined name drug represents the drug-drug pair is documented to have drug-drug interaction in DrugBank database and the drug name with # represents the drug labeled with the ADE in SIDER database.

| drug1 | drug2 | ADE | r_00_ | r_10_ | r_01_ | r_11_ | N | Ω025 [Rank] | -log10(adjusted-FDR) [Rank] | -log10[FDR] [Rank] | $\mathrm{PRR}_{025 D1}$ | $\mathrm{PRR}_{025 D2}$ | $\mathrm{PRR}_{025 D1D2}$ |
| --- | --- | --- | --- | --- | --- | --- | --- | --- | --- | --- | --- | --- | --- |
| PEGFILGRASTIM | PHENYTOIN^#^ | Delirium | 0.0027 | 0.0031 | 0.0049 | 0.5429 | 38 | 4.9747 [1] | 22.7221 [19] | 23.3188 [9] | 1.9341 | 2.0910 | 162.7558 |
| PHENYTOIN^#^ | CARBOPLATIN | Delirium | 0.0027 | 0.0050 | 0.0035 | 0.2713 | 35 | 4.3066 [2] | 13.3545 [46] | 14.7447 [30] | 2.0910 | 1.5340 | 76.0028 |
| PEGFILGRASTIM | VALPROIC ACID^#^ | Delirium | 0.0026 | 0.0031 | 0.0085 | 0.3689 | 38 | 4.2639 [3] | 18.6823 [26] | 14.433 [38] | 1.9341 | 3.2050 | 106.5403 |
| BEVACIZUMAB | PHENYTOIN^#^ | Delirium | 0.0027 | 0.0013 | 0.0048 | 0.1754 | 40 | 4.1906 [4] | 22.3023 [22] | 22.1561 [14] | 0.6918 | 2.0910 | 49.2345 |
| ROFECOXIB | THALIDOMIDE | Delirium | 0.0027 | 0.0022 | 0.0026 | 0.1347 | 26 | 4.0898 [5] | 9.1219 [116] | 14.6289 [35] | 0.8227 | 1.1444 | 34.9943 |
| VALPROIC ACID^#^ | CARBOPLATIN | Delirium | 0.0026 | 0.0085 | 0.0035 | 0.3136 | 37 | 4.0513 [6] | 15.4286 [34] | 11.8894 [53] | 3.2050 | 1.5340 | 89.2514 |
| PREGABALIN^#^ | ROFECOXIB^#^ | Neuropathy | 0.0076 | 0.0179 | 0.0148 | 0.5287 | 83 | 3.9335 [7] | 7.396 [206] | 9.4949 [101] | 2.3263 | 1.9221 | 58.2825 |
| ZIDOVUDINE^#^ | MYCOPHENOLATE MOFETIL^#^ | Neuropathy | 0.0078 | 0.0181 | 0.0095 | 0.6038 | 32 | 3.8718 [8] | 9.0506 [120] | 10.9957 [63] | 2.2673 | 1.2090 | 61.9493 |
| BEVACIZUMAB | VALPROIC ACID^#^ | Delirium | 0.0026 | 0.0013 | 0.0085 | 0.2346 | 38 | 3.8676 [9] | 23.1426 [18] | 18.1864 [16] | 0.6918 | 3.2050 | 66.0302 |
| OLMESARTAN | AMANTADINE | Neuropathy | 0.0079 | 0.0120 | 0.0113 | 0.4459 | 33 | 3.8323 [10] | 1.5069 [2239] | 10.6402 [70] | 1.5402 | 1.8075 | 44.1464 |
| QUININE^#^ | MELOXICAM | Delirium | 0.0027 | 0.0023 | 0.0035 | 0.1517 | 22 | 3.8114 [11] | 7.7893 [182] | 11.7122 [56] | 1.8838 | 1.4280 | 38.3387 |
| FILGRASTIM | PHENYTOIN^#^ | Delirium | 0.0027 | 0.0041 | 0.0051 | 0.1854 | 33 | 3.8094 [12] | 10.291 [81] | 9.9508 [92] | 1.9407 | 2.0910 | 50.6368 |
| NALOXONE | BUDESONIDE | Neuropathy | 0.0079 | 0.0059 | 0.0064 | 0.3621 | 21 | 3.809 [13] | 17.4483 [28] | 13.1979 [45] | 0.8758 | 0.7835 | 32.8251 |
| EFAVIRENZ^#^ | MYCOPHENOLATE MOFETIL^#^ | Neuropathy | 0.0079 | 0.0218 | 0.0094 | 0.6034 | 35 | 3.7685 [14] | 7.935 [173] | 10.202 [85] | 2.8036 | 1.2090 | 62.5101 |
| ALTEPLASE | OMEPRAZOLE^#^ | Neuropathy | 0.0076 | 0.0071 | 0.0196 | 0.4455 | 45 | 3.7653 [15] | 0.4418 [5150] | 11.8268 [55] | 2.5648 | 2.5095 | 45.7570 |
| PEGFILGRASTIM | PAROXETINE^#^ | Neuropathy | 0.0077 | 0.0180 | 0.0146 | 0.4729 | 61 | 3.6966 [16] | 2.118 [1664] | 7.9626 [193] | 2.6096 | 1.8652 | 50.3332 |
| CARBOPLATIN^#^ | VARENICLINE | Neuropathy | 0.0079 | 0.0181 | 0.0025 | 0.5294 | 27 | 3.6936 [17] | 18.4633 [27] | 17.4828 [22] | 2.2121 | 0.3180 | 52.1493 |
| ROFECOXIB | CHLORHEXIDINE | Delirium | 0.0027 | 0.0023 | 0.0029 | 0.1028 | 22 | 3.6766 [18] | 6.9044 [259] | 10.8894 [65] | 0.8227 | 2.2578 | 25.6944 |
| ROFECOXIB^#^ | CEFTRIAXONE | Neuropathy | 0.0077 | 0.0151 | 0.0204 | 0.5179 | 58 | 3.6535 [19] | 1.3528 [2435] | 7.2692 [264] | 1.9221 | 2.8201 | 55.3098 |
| DEXTROMETHORPHAN | CORTISONE ACETATE | Neuropathy | 0.0079 | 0.0120 | 0.0117 | 0.3974 | 31 | 3.6493 [20] | 0.859 [3470] | 9.2204 [110] | 1.8788 | 1.9373 | 38.5905 |
| PHENYLEPHRINE | CORTISONE ACETATE | Neuropathy | 0.0079 | 0.0152 | 0.0116 | 0.4697 | 31 | 3.634 [21] | 1.2474 [2589] | 8.7645 [136] | 2.6395 | 1.9373 | 46.3841 |
| CARBIDOPA | OLMESARTAN | Neuropathy | 0.0079 | 0.0122 | 0.0122 | 0.4143 | 29 | 3.5928 [22] | 7.3858 [207] | 8.6968 [141] | 1.6155 | 1.5402 | 40.0122 |
| ALTEPLASE | ATROPINE | Neuropathy | 0.0079 | 0.0140 | 0.0144 | 0.5088 | 29 | 3.5902 [23] | 0.0542 [19074] | 8.1403 [175] | 2.5648 | 2.2515 | 50.3031 |
| SUCRALFATE | METAXALONE | Delirium | 0.0027 | 0.0037 | 0.0017 | 0.2000 | 16 | 3.5857 [24] | 9.4187 [107] | 10.7122 [68] | 1.6663 | 1.2557 | 47.8868 |
| CONJUGATED ESTROGENS | DEXTROMETHORPHAN | Neuropathy | 0.0079 | 0.0096 | 0.0120 | 0.3263 | 31 | 3.5799 [25] | 3.1298 [959] | 9.0985 [116] | 1.1881 | 1.8788 | 31.1922 |
| MYCOPHENOLATE MOFETIL^#^ | RALTEGRAVIR^#^ | Neuropathy | 0.0079 | 0.0095 | 0.0176 | 0.4384 | 32 | 3.5735 [26] | 9.124 [115] | 8.8447 [132] | 1.2090 | 2.4462 | 43.1474 |
| CONJUGATED ESTROGENS | CORTISONE ACETATE | Neuropathy | 0.0079 | 0.0097 | 0.0127 | 0.3750 | 27 | 3.5718 [27] | 5.731 [363] | 8.9393 [127] | 1.1881 | 1.9373 | 35.5112 |
| ESZOPICLONE | ROFECOXIB^#^ | Neuropathy | 0.0077 | 0.0085 | 0.0155 | 0.3678 | 32 | 3.5577 [28] | 6.591 [279] | 9.0964 [117] | 1.1537 | 1.9221 | 35.6361 |
| ROFECOXIB | GRANISETRON | Delirium | 0.0027 | 0.0024 | 0.0035 | 0.1798 | 16 | 3.5553 [29] | 10.2715 [82] | 9.5544 [100] | 0.8227 | 1.4602 | 42.8081 |
| DULOXETINE | ROFECOXIB^#^ | Neuropathy | 0.0076 | 0.0200 | 0.0148 | 0.4229 | 85 | 3.5546 [30] | 10.9655 [69] | 6.4921 [348] | 2.6122 | 1.9221 | 45.9861 |
| OLMESARTAN | LEVODOPA^#^ | Neuropathy | 0.0079 | 0.0122 | 0.0106 | 0.3488 | 30 | 3.5469 [31] | 7.3962 [205] | 8.6596 [143] | 1.5402 | 1.3981 | 33.3499 |
| CETIRIZINE | LEVODOPA | Neuropathy | 0.0078 | 0.0155 | 0.0101 | 0.3592 | 37 | 3.5289 [32] | 0.3621 [5716] | 8.4342 [157] | 1.9435 | 1.3981 | 35.4248 |
| SUCRALFATE | ROSUVASTATIN | Delirium | 0.0027 | 0.0029 | 0.0015 | 0.0925 | 21 | 3.5208 [33] | 7.9941 [169] | 12.0083 [51] | 1.6663 | 0.5564 | 22.8526 |
| IBUPROFEN^#^ | TINZAPARIN | Neuropathy | 0.0078 | 0.0154 | 0.0112 | 0.4727 | 26 | 3.5191 [34] | 0.0537 [19226] | 8.0496 [186] | 1.8997 | 2.1063 | 45.6265 |
| ROFECOXIB^#^ | LAMOTRIGINE^#^ | Neuropathy | 0.0077 | 0.0155 | 0.0081 | 0.3271 | 35 | 3.5168 [35] | 11.8226 [59] | 9.0391 [122] | 1.9221 | 1.0212 | 31.8124 |
| CLONIDINE | AMANTADINE | Neuropathy | 0.0078 | 0.0221 | 0.0109 | 0.5147 | 35 | 3.5167 [36] | 0.8837 [3405] | 7.767 [206] | 2.7346 | 1.8075 | 52.1499 |
| QUININE^#^ | TIOTROPIUM | Delirium | 0.0027 | 0.0030 | 0.0020 | 0.1061 | 19 | 3.5034 [37] | 4.7523 [514] | 10.1135 [87] | 1.8838 | 0.7381 | 25.7572 |
| VALDECOXIB^#^ | TELMISARTAN^#^ | Neuropathy | 0.0079 | 0.0093 | 0.0072 | 0.3462 | 18 | 3.4979 [38] | 10.6144 [76] | 9.1512 [114] | 1.1240 | 0.8844 | 30.3932 |
| MOMETASONE | LEVODOPA^#^ | Neuropathy | 0.0079 | 0.0188 | 0.0106 | 0.4762 | 30 | 3.4971 [39] | 1.9748 [1757] | 7.8996 [196] | 2.3642 | 1.3981 | 46.9002 |
| TRASTUZUMAB | TIOTROPIUM | Neuropathy | 0.0079 | 0.0300 | 0.0051 | 0.5443 | 43 | 3.4926 [40] | 9.0462 [121] | 13.0453 [47] | 3.7995 | 0.6675 | 56.7847 |
| ALTEPLASE | RAMIPRIL^#^ | Neuropathy | 0.0078 | 0.0141 | 0.0133 | 0.4394 | 29 | 3.4885 [41] | 0.0683 [15941] | 7.6073 [226] | 2.5648 | 1.6365 | 42.6940 |
| BUPIVACAINE | PREDNISONE^#^ | Delirium | 0.0027 | 0.0042 | 0.0028 | 0.2273 | 15 | 3.485 [42] | 11.2454 [67] | 8.6271 [146] | 2.0987 | 0.9646 | 54.0550 |
| FLUTICASONE PROPIONATE | AMANTADINE | Neuropathy | 0.0078 | 0.0128 | 0.0089 | 0.2460 | 46 | 3.4751 [43] | 1.7166 [2001] | 8.6364 [144] | 1.6081 | 1.8075 | 24.4347 |
| CARBIDOPA | CETIRIZINE | Neuropathy | 0.0078 | 0.0116 | 0.0155 | 0.3750 | 36 | 3.4708 [44] | 0.1599 [8870] | 7.644 [221] | 1.6155 | 1.9435 | 36.9677 |
| BUPIVACAINE | ZOLEDRONATE | Delirium | 0.0027 | 0.0042 | 0.0034 | 0.2459 | 15 | 3.4567 [45] | 10.361 [80] | 8.2343 [171] | 2.0987 | 1.1792 | 58.8019 |
| CYCLIZINE | CLARITHROMYCIN | Delirium | 0.0027 | 0.0070 | 0.0100 | 0.4118 | 21 | 3.4523 [46] | 4.7152 [528] | 5.5114 [534] | 5.8248 | 3.6446 | 110.1161 |
| MOXIFLOXACIN^#^ | DOCETAXEL^#^ | Neuropathy | 0.0077 | 0.0190 | 0.0301 | 0.5714 | 96 | 3.4485 [47] | 0.0516 [19873] | 4.2733 [1020] | 2.7457 | 4.0170 | 64.0834 |
| PAROXETINE^#^ | TINZAPARIN | Neuropathy | 0.0078 | 0.0152 | 0.0112 | 0.4333 | 26 | 3.4471 [48] | 0.0527 [19561] | 7.618 [223] | 1.8652 | 2.1063 | 41.3985 |
| TRASTUZUMAB | PAROXETINE^#^ | Neuropathy | 0.0077 | 0.0254 | 0.0141 | 0.4414 | 98 | 3.4464 [49] | 2.4143 [1350] | 5.6073 [512] | 3.7995 | 1.8652 | 48.6757 |
| CARBIDOPA | MOMETASONE | Neuropathy | 0.0079 | 0.0121 | 0.0188 | 0.4839 | 30 | 3.4437 [50] | 1.4641 [2295] | 7.1518 [276] | 1.6155 | 2.3642 | 47.7475 |
| PEGFILGRASTIM | BEVACIZUMAB | Delirium | 0.0027 | 0.0029 | 0.0012 | 0.0511 | 43 | 3.441 [51] | 16.6822 [31] | 16.2976 [25] | 1.9341 | 0.6918 | 14.1947 |
| ROSIGLITAZONE | BIMATOPROST | Neuropathy | 0.0079 | 0.0086 | 0.0048 | 0.2951 | 18 | 3.4377 [52] | 10.8976 [71] | 10.4045 [78] | 1.0349 | 0.7282 | 25.5408 |
| OLMESARTAN | HALOPERIDOL | Neuropathy | 0.0078 | 0.0116 | 0.0258 | 0.5263 | 40 | 3.4311 [53] | 5.5694 [382] | 6.7905 [312] | 1.5402 | 3.2299 | 54.2751 |
| ESOMEPRAZOLE | CYCLIZINE | Delirium | 0.0027 | 0.0028 | 0.0087 | 0.2754 | 19 | 3.4214 [54] | 8.0377 [165] | 8.158 [174] | 0.9941 | 5.8248 | 69.7087 |
| ROSUVASTATIN^#^ | TINZAPARIN | Neuropathy | 0.0079 | 0.0111 | 0.0129 | 0.4231 | 22 | 3.4181 [55] | 0.0807 [14147] | 7.684 [217] | 1.3500 | 2.1063 | 39.2969 |
| GEMCITABINE | ROFECOXIB | Delirium | 0.0027 | 0.0021 | 0.0024 | 0.1261 | 15 | 3.4159 [56] | 10.4108 [79] | 9.4559 [106] | 0.8139 | 0.8227 | 29.1456 |
| BUPIVACAINE | WARFARIN | Delirium | 0.0027 | 0.0042 | 0.0041 | 0.2632 | 15 | 3.415 [57] | 8.9722 [125] | 7.7721 [205] | 2.0987 | 1.4521 | 63.2471 |
| SUCRALFATE | EZETIMIBE | Delirium | 0.0027 | 0.0033 | 0.0015 | 0.0990 | 19 | 3.3966 [58] | 8.9313 [127] | 10.762 [67] | 1.6663 | 0.5943 | 23.9723 |
| VALDECOXIB^#^ | ACYCLOVIR | Neuropathy | 0.0078 | 0.0082 | 0.0332 | 0.5968 | 37 | 3.3947 [59] | 6.1905 [313] | 8.1029 [178] | 1.1240 | 4.0724 | 62.0710 |
| METAXALONE | ROSUVASTATIN | Delirium | 0.0027 | 0.0017 | 0.0016 | 0.1060 | 16 | 3.3886 [60] | 12.0456 [55] | 11.3002 [60] | 1.2557 | 0.5564 | 24.7431 |
| CELECOXIB^#^ | VANCOMYCIN | Neuropathy | 0.0077 | 0.0156 | 0.0203 | 0.3675 | 111 | 3.3868 [61] | 4.9348 [475] | 5.2175 [622] | 2.0992 | 3.0377 | 40.5446 |
| RAMIPRIL^#^ | ATROPINE | Neuropathy | 0.0078 | 0.0132 | 0.0126 | 0.3214 | 36 | 3.3746 [62] | 2.9932 [1005] | 7.1013 [281] | 1.6365 | 2.2515 | 31.3452 |
| CONJUGATED ESTROGENS | PHENYLEPHRINE | Neuropathy | 0.0079 | 0.0096 | 0.0154 | 0.3333 | 31 | 3.3739 [63] | 3.6248 [778] | 7.58 [229] | 1.1881 | 2.6395 | 31.9111 |
| LEFLUNOMIDE^#^ | PANCURONIUM | skin pigmentation disorder | 0.0214 | 0.0486 | 0.0340 | 0.9231 | 48 | 3.3571 [64] | 3.4924 [813] | 6.5361 [344] | 2.1843 | 2.9954 | 40.4508 |
| GEMCITABINE | DULOXETINE | Delirium | 0.0027 | 0.0020 | 0.0056 | 0.1868 | 17 | 3.3538 [65] | 10.6277 [74] | 9.0625 [119] | 0.8139 | 1.9728 | 45.1643 |
| TETRACYCLINE | DIAZEPAM | Neuropathy | 0.0078 | 0.0166 | 0.0213 | 0.4938 | 40 | 3.3387 [66] | 0.0187 [52732] | 5.2848 [601] | 4.1372 | 2.6486 | 50.5601 |
| AMPICILLIN | GEMCITABINE^#^ | Neuropathy | 0.0078 | 0.0418 | 0.0203 | 0.8136 | 48 | 3.3384 [67] | 0.0192 [51123] | 3.6603 [1408] | 5.7348 | 2.6177 | 91.9059 |
| CONJUGATED ESTROGENS | CINACALCET | Neuropathy | 0.0079 | 0.0098 | 0.0063 | 0.2951 | 18 | 3.3357 [68] | 3.9603 [673] | 8.7212 [140] | 1.1881 | 0.9996 | 25.5408 |
| OXYBUTYNIN | CEFUROXIME | Neuropathy | 0.0079 | 0.0191 | 0.0207 | 0.5690 | 33 | 3.3301 [69] | 0.0191 [51411] | 5.1302 [667] | 2.4807 | 2.8649 | 58.0363 |
| ALTEPLASE | FENTANYL^#^ | Neuropathy | 0.0077 | 0.0103 | 0.0176 | 0.3363 | 38 | 3.3234 [70] | 0.0902 [13082] | 6.8962 [298] | 2.5648 | 2.1887 | 33.1271 |
| PEGFILGRASTIM | FILGRASTIM | Delirium | 0.0027 | 0.0027 | 0.0033 | 0.0527 | 45 | 3.3199 [71] | 6.7637 [269] | 8.0506 [184] | 1.9341 | 1.9407 | 14.7484 |
| QUININE^#^ | BISOPROLOL | Delirium | 0.0027 | 0.0028 | 0.0038 | 0.1031 | 20 | 3.3139 [72] | 3.3631 [860] | 7.6253 [222] | 1.8838 | 1.4261 | 25.2713 |
| TETRACYCLINE | ESCITALOPRAM^#^ | Delirium | 0.0027 | 0.0007 | 0.0055 | 0.2143 | 15 | 3.3108 [73] | 10.9939 [68] | 11.3799 [59] | 2.3461 | 1.9282 | 50.7768 |
| ZOLEDRONATE | ROFECOXIB | Delirium | 0.0027 | 0.0030 | 0.0021 | 0.0486 | 41 | 3.306 [74] | 8.5685 [143] | 10.1129 [88] | 1.1792 | 0.8227 | 13.4005 |
| PAROXETINE^#^ | LAPATINIB^#^ | Neuropathy | 0.0078 | 0.0151 | 0.0117 | 0.3529 | 30 | 3.3053 [75] | 5.571 [381] | 6.6478 [334] | 1.8652 | 1.5599 | 33.7730 |
| PEGFILGRASTIM | FENOFIBRATE | Neuropathy | 0.0078 | 0.0208 | 0.0157 | 0.5263 | 30 | 3.3031 [76] | 2.8258 [1100] | 5.4935 [536] | 2.6096 | 1.9453 | 52.4992 |
| TRASTUZUMAB | MOXIFLOXACIN^#^ | Neuropathy | 0.0078 | 0.0297 | 0.0215 | 0.6479 | 46 | 3.3012 [77] | 1.1703 [2727] | 3.7649 [1341] | 3.7995 | 2.7457 | 69.6727 |
| ZOLPIDEM^#^ | DROPERIDOL | Neuropathy | 0.0076 | 0.0276 | 0.0133 | 0.5000 | 46 | 3.2972 [78] | 0.0469 [21509] | 5.2269 [621] | 3.4816 | 6.5154 | 52.0335 |
| FENTANYL^#^ | METHAMPHETAMINE | Neuropathy | 0.0077 | 0.0178 | 0.0022 | 0.4074 | 22 | 3.2966 [79] | 0.052 [19748] | 13.9666 [40] | 2.1887 | 3.9370 | 37.6799 |
| SUCRALFATE | CARISOPRODOL | Delirium | 0.0027 | 0.0035 | 0.0033 | 0.1278 | 17 | 3.2952 [80] | 3.2963 [892] | 7.3768 [252] | 1.6663 | 1.3689 | 30.4335 |
| GEMCITABINE^#^ | DRONABINOL | Neuropathy | 0.0078 | 0.0205 | 0.0169 | 0.4423 | 46 | 3.2904 [81] | 0.905 [3350] | 4.9914 [715] | 2.6177 | 4.3981 | 45.5054 |
| SALBUTAMOL^#^ | OXYMORPHONE | Neuropathy | 0.0078 | 0.0143 | 0.0053 | 0.3571 | 20 | 3.2875 [82] | 2.7728 [1132] | 9.3696 [108] | 1.7605 | 1.3808 | 32.0623 |
| METRONIDAZOLE | ALUMINUM HYDROXIDE | Delirium | 0.0027 | 0.0058 | 0.0048 | 0.2941 | 15 | 3.2836 [83] | 4.5382 [558] | 6.5406 [343] | 2.0485 | 2.8085 | 71.3425 |
| BUPROPION^#^ | METHAMPHETAMINE | Neuropathy | 0.0078 | 0.0181 | 0.0000 | 0.3898 | 23 | 3.2731 [84] | 35.038 [9] | 22.2097 [10] | 2.2115 | 3.9370 | 36.1433 |
| METOLAZONE | METAXALONE | Delirium | 0.0027 | 0.0075 | 0.0019 | 0.2727 | 15 | 3.2687 [85] | 2.5664 [1246] | 8.5607 [149] | 3.0039 | 1.2557 | 65.7327 |
| QUININE | CYCLOPHOSPHAMIDE^#^ | Neuropathy | 0.0077 | 0.0273 | 0.0266 | 0.6949 | 41 | 3.2643 [86] | 0.0253 [38115] | 3.3397 [1701] | 3.8851 | 3.3156 | 74.8984 |
| ESOMEPRAZOLE | CEFADROXIL | Neuropathy | 0.0077 | 0.0157 | 0.0076 | 0.3492 | 22 | 3.2642 [87] | 0.0078 [189286] | 7.5528 [232] | 1.9475 | 4.7051 | 31.8028 |
| ALLOPURINOL^#^ | DESONIDE | Neuropathy | 0.0078 | 0.0197 | 0.0097 | 0.4444 | 24 | 3.2613 [88] | 0.0173 [57736] | 6.7305 [323] | 2.4072 | 4.5376 | 42.0837 |
| SIMVASTATIN^#^ | MITOXANTRONE | Neuropathy | 0.0077 | 0.0157 | 0.0058 | 0.2917 | 28 | 3.2594 [89] | 8.7618 [130] | 9.3125 [109] | 1.9684 | 1.2307 | 27.2494 |
| PAROXETINE^#^ | DOCETAXEL^#^ | Neuropathy | 0.0077 | 0.0135 | 0.0281 | 0.3865 | 143 | 3.2586 [90] | 0.1097 [11449] | 4.4191 [936] | 1.8652 | 4.0170 | 43.5161 |
| ALTEPLASE | ZOLEDRONATE | Neuropathy | 0.0074 | 0.0057 | 0.0440 | 0.6234 | 48 | 3.2579 [91] | 0.2445 [7034] | 9.6925 [96] | 2.5648 | 5.6373 | 66.8983 |
| ACETAMINOPHEN | THIETHYLPERAZINE | Neuropathy | 0.0072 | 0.0207 | 0.0068 | 0.4211 | 24 | 3.2539 [92] | 0.0245 [39376] | 7.71 [213] | 2.7451 | 10.8307 | 39.6219 |
| WARFARIN | TETRACYCLINE | Delirium | 0.0027 | 0.0041 | 0.0007 | 0.1471 | 15 | 3.2537 [93] | 14.7344 [38] | 11.1637 [61] | 1.4521 | 2.3461 | 34.1982 |
| CARVEDILOL | NALOXONE | Neuropathy | 0.0078 | 0.0150 | 0.0055 | 0.3077 | 24 | 3.253 [94] | 13.0354 [49] | 9.4001 [107] | 1.8283 | 0.8758 | 28.1420 |
| NATEGLINIDE^#^ | HEPARIN | Neuropathy | 0.0079 | 0.0137 | 0.0109 | 0.3538 | 23 | 3.2525 [95] | 1.5827 [2147] | 6.7122 [327] | 2.0068 | 1.3255 | 32.5041 |
| SUCRALFATE | MEMANTINE^#^ | Delirium | 0.0027 | 0.0033 | 0.0113 | 0.3214 | 18 | 3.2503 [96] | 0.3167 [6144] | 6.5986 [338] | 1.6663 | 4.0622 | 81.5437 |
| ZOLEDRONATE | RIZATRIPTAN | skin pigmentation disorder | 0.0216 | 0.0214 | 0.0124 | 0.4364 | 24 | 3.2479 [97] | 9.4423 [106] | 10.5969 [72] | 0.9568 | 0.7672 | 15.3156 |
| METAXALONE | TERIPARATIDE | Delirium | 0.0027 | 0.0022 | 0.0011 | 0.1102 | 14 | 3.2475 [98] | 14.0579 [40] | 10.2549 [82] | 1.2557 | 0.3933 | 24.9570 |
| METOPROLOL | CEFADROXIL | Neuropathy | 0.0076 | 0.0175 | 0.0075 | 0.3793 | 22 | 3.2418 [99] | 0.0082 [175160] | 7.4389 [244] | 2.1934 | 4.7051 | 34.8179 |
| TICLOPIDINE^#^ | SALMETEROL | Neuropathy | 0.0079 | 0.0058 | 0.0095 | 0.2963 | 16 | 3.2373 [100] | 8.8509 [129] | 8.3883 [161] | 0.9137 | 1.1436 | 25.0571 |
